# Multi-omic analysis reveals enriched pathways associated with COVID-19 and COVID-19 severity

**DOI:** 10.1371/journal.pone.0267047

**Published:** 2022-04-25

**Authors:** Danika Lipman, Sandra E. Safo, Thierry Chekouo

**Affiliations:** 1 Department of Mathematics and Statistics, University of Calgary, Calgary, Alberta, Canada; 2 Division of Biostatistics, University of Minnesota, Minneapolis, Minnesota, United States of America; 3 Department of Biochemistry and Molecular Biology, University of Calgary, Calgary, AB, Canada; Pacific Northwest National Laboratory, UNITED STATES

## Abstract

COVID-19 is a disease characterized by its seemingly unpredictable clinical outcomes. In order to better understand the molecular signature of the disease, a recent multi-omics study was done which looked at correlations between biomolecules and used a tree- based machine learning approach to predict clinical outcomes. This study specifically looked at patients admitted to the hospital experiencing COVID-19 or COVID-19 like symptoms. In this paper we examine the same multi-omics data, however we take a different approach, and we identify *stable* molecules of interest for further pathway analysis. We used stability selection, regularized regression models, enrichment analysis, and principal components analysis on proteomics, metabolomics, lipidomics, and RNA sequencing data, and we determined key molecules and biological pathways in disease severity, and disease status. In addition to the individual omics analyses, we perform the integrative method Sparse Multiple Canonical Correlation Analysis to analyse relationships of the different view of data. Our findings suggest that COVID-19 status is associated with the cell cycle and death, as well as the inflammatory response. This relationship is reflected in all four sets of molecules analyzed. We further observe that the metabolic processes, particularly processes to do with vitamin absorption and cholesterol are implicated in COVID-19 status and severity.

## 1. Introduction

As of July 13, 2021 4,103,278 people have died as a result of the severe acute respiratory syndrome coronavirus 2 (SARS-CoV-2), more commonly known as COVID-19 (coronavirus disease of 2019). Since the first outbreak of COVID-19 was reported in 2019, the world has experienced a vast change in lifestyle in response to the unpredictable nature of the disease. One of the primary characteristics of the disease which concerns health experts worldwide is the varying severity individuals experience, which correlates with distinct genetic and physiological conditions of the infected patients. Deaths from the disease worldwide have been related to acute respiratory distress syndrome (ARDS), a serious lung injury which allows fluids to leak into the lungs [1]. In order to better understand the molecular signatures of COVID-19, we performed an analysis of multi-omics data from patients experiencing COVID-19 or COVID-19 like symptoms admitted to the hospital for ARDS.

A recent multi-omics study by Overmyer et al., 2020 [2] quantified transcripts, proteins, metabolites, and lipids from patients with COVID-19 and patients experiencing COVID-19 like symptoms. These molecules were then associated with clinical outcomes including comorbidities, ICU (intensive care unit) status, and disease severity through correlation analysis and machine learning techniques. From these analyses, the unique signature of the disease was apparent in a dysregulated lipid transport system, complement activation, and neutrophil activation. We have taken an alternative approach in analyzing this dataset by identifying *stable* molecules of interest for further enrichment analysis. More specifically, in order to determine key molecules in COVID-19 status and severity, we employed stability selection [3] and regularized regression models to each set of molecules. These molecules were further analyzed through enrichment analysis, which revealed key pathways enriched in COVID-19. The significant pathways were then summarized through their first two principal components, which were then used as predictors in multivariate regression models. From these multivariate regression models, we were able to assess the significance of the pathways in COVID-19 status and severity. In addition to the individual analyses of each view we take the integrative approach Sparse Multiple Canonical Correlation Analysis (smCCA) to assess relationships between the views of data [4].

## 2. Materials and methods

### 2.1 Dataset

The data used for this multi-omic analysis was collected from April 6, 2020, through May 1, 2020, by Overmyer et al., 2020. A total of 128 patients experiencing respiratory issues were admitted to the Albany Medical Center in Albany, NY and had blood taken and clinical data collected. A summary of the clinical variables is provided in S1 Table in S1 File. After the blood samples were taken it was determined which patients had the SARS-CoV-2 infection and resulted in 102 patients testing positive for COVID-19, and the remaining 26 patients testing negative. The data from these patients were used to explore the possible correlation of certain biomarkers with status and severity of COVID-19. The blood samples collected were used for multiple omics analyses. RNAseq was performed on leukocytes isolated from the blood samples. From the blood plasma, mass spectrometry (MS) technology was used to identify and quantify proteins, lipids and metabolites. The data were filtered in two layers. Any molecules which were not significant in either disease status or severity at an alpha of 0.1 were removed from the sample. Following this first layer of filtering, low variance molecules were removed. A more detailed description of the filtering process for these datasets is provided in the methodology section. One of the main goals of our paper is to determine which molecules and molecular pathways are key determinants in disease severity. Two methods were used to measure disease severity in the Overmyer et al., 2020 paper. These methods were the World Health Organization (WHO) 0–8 disease specific scale where 8 denotes death, as well as a score out of 45 indicating the number of hospital free days (HFD-45). A HFD-45 value of 0 indicates the individual was still admitted in the hospital after 45 days, or that the individual died. As mentioned in the Overmyer et al., 2020 paper, the scores give comparable outcomes. However, the HFD-45 measurement is favoured as it is more granular and not a disease specific measurement hence it is easily applied to patients without COVID-19. For the main analyses in this paper, only clinical covariates which were present in all of the samples were used. Specifically, we focus on the Charlson comorbidity index (CCI) score, age and sex. The CCI score is a score to assess the comorbidities of a patient based on the number and severity of comorbid conditions, with higher scores indicating more comorbidities and higher severity. Comorbidities have been shown to be strongly related to COVID-19 outcomes, so this is crucial to fit in the models [5]. Age has also been shown to have a significant effect on the disease severity [6] so models were adjusted to incorporate age. The initial dataset contains 18,212 genes, 517 proteins, 111 molecules from metabolomics analysis, and 3,357 lipids, which were filtered as discussed in the following section.

### 2.2 Filtering the data

The omics data were normalized and transformed with a log base 2. Further details on normalization and initial quality control filtering are available in the Overmyer et al., 2020 paper. For this paper we used the normalized data which passed quality control. These 517 proteins, 111 molecules from metabolomics analysis, and 3,357 lipids were read in from the Sqlite database [7]. Due to some missing clinical data critical to the study, some patients were excluded resulting in 99 patients with COVID-19 and 24 patients without COVID-19. For this study the raw RNAseq data on 18,212 genes was read from the National Center for Biotechnology Information. Once reading in the data we apply our own filtering methods. All genes which were missing in over 70% of the samples were removed from the dataset and 15740 genes remained. Any remaining missing values were imputed via the K-nearest neighbourhood algorithm (k = 11). The algorithm is easily implemented using the *Impute* package in R [8]. The resulting log base 2 transformed data were filtered via univariate regression at the significance level alpha of 0.1. Any molecule which was not statistically significantly associated with COVID-19 or severity was removed from the data. To determine significance with COVID-19, logistic regression models were fit using COVID-19 status as the outcome. Linear regression models were fit using HFD-45 as a continuous response to determine significance with severity. Each molecule was tested for significance using the likelihood-ratio test adjusting for age and sex. This filtering method resulted in 14499 genes, 80 molecules from metabolomics analysis, 352 proteins, and 2031 lipids. Following this layer of filtering, the molecules with low variation were removed from the analysis. The threshold for low variation was determined separately for each molecule type by analyzing a histogram of the variances. A visual of this filtering process is provided in S1 Fig in S1 File. Following filtering, the dataset to be analyzed consists of 5800 genes, 72 molecules from metabolomics analysis, 264 proteins, and 1015 lipids. Some of the molecules remaining after the filtering process were unidentified, specifically we were left with unidentified lipids and metabolites. Of the 863 unidentified lipids we were able to identify 693 using LIPID MAPS® comprehensive classification system for lipids, which uses retention time and mass per charge (m/z) to make an identification, and we allowed a tolerance of 0.05 [9]. Unfortunately, none of the 31 unknown molecules from metabolomics were annotated in the original Overmeyer et al. paper and could not be identified via a search of the HMDB database. Any unknown molecules were excluded from enrichment analysis.

### 2.3 Stability selection

In order to select the biomolecules we would like to analyze, we used the elastic net regularization method coupled with stability selection [10] with error control as implemented in the *stabs* package in R. Stability selection is a resampling method to control for type 1 error [29]. In order for a variable to be included in the selection process, it must be selected over a set threshold proportion of the subsamples. Typically, this threshold is set between 0.6 and 0.9, and in the study, we specifically set the threshold to 0.6. It should be noted that increasing the threshold had little to no effect on the results. In addition to this tuning parameter, stability selection also requires either a restriction on the per-family-error rate or the parameter which specifies the average number of variables to be selected at each subsampling iteration. This parameter can easily be calculated to set the error rate at a fixed level and will be dependent on the total number of molecules which we are selecting from and the specified threshold. In this case the parameter was specified to set the family wise error rate to 0.05. For each of the four molecule types, stability selection was used three times. Stability selection was used with i) all patients and COVID-19 status as the response, ii) all patients with severity measured by HFD-45 as the outcome, and iii) only patients with COVID-19 and HFD-45 as the outcome. The groups of selected molecules are then independently inspected via enrichment analysis.

### 2.4 Enrichment analysis

To further examine the selected biomolecules, we performed enrichment analyses. For the metabolomics, proteomics, and RNAseq data, the Ingenuity Pathway Analysis (IPA) [11] software was used. The lipid set enrichment analyses were performed with Lipid Pathway Enrichment Analysis (LIPEA), which is an online tool specifically designed for the analysis of lipidomics data. Both of these methodologies are similar in how they operate. From the output from IPA, we will focus on the predicted pathways that are enriched (top canonical pathways) and a prediction of affected biology (top diseases and biological functions). IPA calculates the p-values using a right-tailed Fisher’s exact test to determine whether molecules indicate a pathway is significant hence the p-values provide insight into the probability the molecules were selected randomly. The LIPEA software gives less information than IPA. The enriched pathways are given along with the p-values of their significance, however, LIPEA uses the over-representation analysis methodology (ORA) to calculate p-values and determine enriched pathways [12].

### 2.5 Associating molecules with clinical outcomes

Following enrichment analysis, we performed further analysis to determine pathways strongly associated with clinical outcomes. Specifically, we performed principal components analysis (PCA) on the molecules in enriched pathways. Principal components (PCs) are useful because they are orthogonal and hence make sure we are getting uncorrelated modes of variation. With these principal components we are able to assess the correlation with clinical covariates and fit regression models to assess how well the enriched pathways are able to predict clinical outcomes. The first two PCs of the significant pathways were fit as covariates in regression models. The pathways selected for COVID-19 status were fit with disease status as the outcome in a logistic regression model adjusting for age and sex. The pathways selected for disease severity for all patients were fit in a model with HFD-45 as the response adjusting for age and sex. The process is the same for molecules selected specifically for COVID-19 severity, however the models are fit using the subset of patients which tested positive for COVID-19.

### 2.6 Unsupervised integrative analysis

In order to better assess the relationships between the views of data we implement the unsupervised method Sparse Multiple Canonical Correlation Analysis (smCCA) [4]. smCCA is an integrative method that works to determine key features in multiple views of data, while maximizing the correlation across the views. This method uses penalized matrix decomposition to identify sparse linear combinations of the correlated datasets and an L1 penalty to induce sparsity on coefficients. Inducing sparsity allows us to determine a smaller amount of key molecules that captured the correlation of the multi-view data. In order to choose the parameters for the L1 penalty, we permute the data 100 times and smCCA is performed on each permutation, and a Fisher z-statistic is used to select the optimal parameters. Once the penalty parameters have been selected, smCCA is performed on the four views of data using 100 iterations.

## 3. Results

In Section 5.1, we first discuss the results with respect to COVID-19 status, while the next two sections (Section 5.2 and 5.3) focus on the results related to disease severity. Sections 5.2 and 5.3 include results found from analysis performed on data from all patients, and those with COVID-19 only, respectively. The final section (Section 5.4) presents the results of the smCCA analysis. In addition, violin plots of the top stability selected genes and proteins for COVID-19 status are provided in the S2, S3 Figs in S1 File.

### 3.1 COVID-19 status

**Fig 1:**
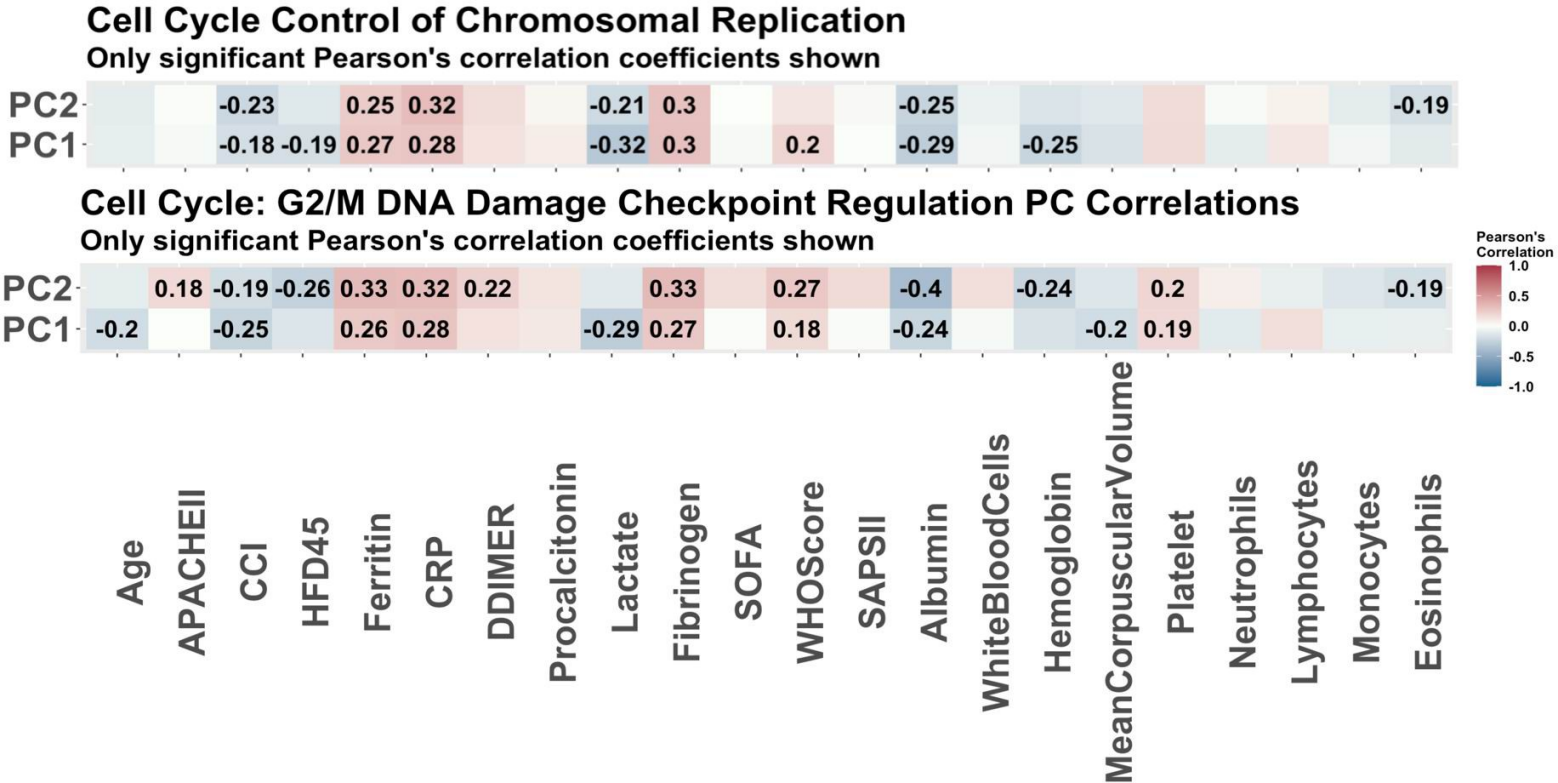
The Pearson correlations of principal components of pathways associated with disease status. The Pearson correlations of clinical variables and principal components used to summarize the enriched pathways in COVID-19 as predicted by IPA. These are the pathways predicted to be enriched based on 16 genes determined to be associated with COVID-19 via stability selection. Only the correlations which were significant (p-value<0.05) are reported. Some of the strongest correlations are with ferritin, CRP, and lactate.

**Fig 2:**
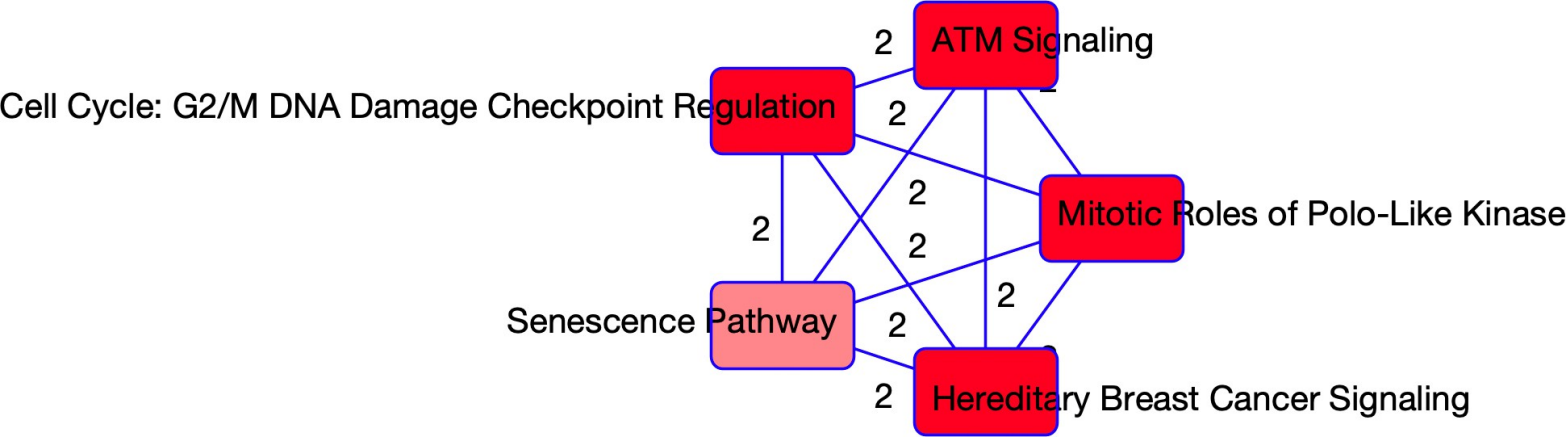
Overlapping networks associated with COVID-19. Visual of the overlapping networks enriched in COVID-19 as determined from the RNAseq data. The nodes represent the networks and the edges represent the overlapping genes between the networks. The edge labels give the number of overlapping molecules between the networks.

Stability selection on the RNA seq data identified 16 stable genes that were associated with COVID-19 status (refer to S2A Table in S1 File). A core analysis using the Ingenuity Pathway Analysis (IPA) software on these 16 genes revealed the top five enriched canonical pathways, and top ten predicted effects on biology. This output is summarized in Table 1. Principal components analysis was performed on each pathway and the first two principal components (PCs) were used to summarize the pathways. A Pearson’s correlation matrix displays how the different PCs relate to some of our clinical covariates. The heatmaps of these correlation matrices are available in Fig 1 where we notice that the PCs are significantly correlated with multiple clinical covariates. This correlation is especially evident in the fibrinogen measurements (mg/dL). Fibrinogen is a clotting factor protein which plays a key role in blood clot formation [13]. In order to assess which pathways are significantly associated with disease status, we additionally fit multivariate logistic regression models with COVID-19 status as the response and PCs as predictors. We adjusted the models for age, sex and Charlson score. In Table 2 the summaries for each regression model are supplied. We only focused on the two first pathways as genes in other pathways belong also to those two pathways, and the last pathway only contains one molecule. Refer to Table 1 to see which genes were selected from each pathway. From the regression models, the PCs are significant predictors of COVID-19 status for all of the selected pathways. Notably, the top two pathways are involved in the regulation of the cell cycle. Other than the G2/M DNA damage checkpoint regulation and control of chromosomal replication pathways, we find that the mitotic roles of Polo-Like Kinase pathway are also enriched. This pathway plays a role in cell separation. The ATM-signaling pathway is also enriched which plays a role in activating the DNA damage checkpoint. Together, these findings suggest that the most unique aspects of COVID-19 are its effects on the cell cycle, which aligns with a study performed on Vero E6 cells that further explores dysregulation of the cell cycle [14]. A visual of the overlapping networks is available in Fig 2 where we observe overlapping genes between all the networks.

**Table 1:** Enrichment analysis of genes associated with COVID-19 status.

Top Diseases and Biological Functions		
	**P-value range**	**Selected Molecules**
Connective Tissue Disorders	4.04E-03–1.18E-05	*CDC45*, *CDC6*, *TYMS *
Developmental Disorder	4.04E-03–1.18E-05	*CDC45*, *CDC6*, *TYMS*, *CCNB1*, *OBSL1*
Gastrointestinal Disease	1.21E-02–1.18E-05	*CCNB1*, *CDC25C*, *CDC45*, *CDC6*, *DEPDC1B DIAPH3*, *GINS2*, *MCM10*, *OBSL1*, *PBK*, *TYMS*
Organismal Injury and Abnormalities	1.21E-02–1.18E-05	*CCNB1*, *CDC25C*, *CDC45*, *CDC6*, *DEPDC1B*, *DIAPH3*, *FHL2*, *GINS2*, *MCM10*, *OBSL1*, *PBK*, *TYMS*
Skeletal and Muscular Disorders	7.57E-03- 1.18E-05	*CDC45*, *CDC6*, *TYMS*, *DIAPH3*
Cell Cycle	1.14E-02–5.08E-07	*CCNB1*, *CDC25C*, *CDC45*, *CDC6*, *DIAPH3*, *FHL2*, *MCM10*, *OBSL1*, *TYMS*
DNA Replication, Recombination, and Repair	9.40E-03–1.51E-05	*CCNB1*, *CDC6*, *CDC45*, *MCM10*, *PBK*
Cell-To-Cell Signalling and Interaction	4.04E-03–8.80E-05	*CCNB1*, *TYMS*
Cell Death and Survival	1.01E-02–1.24E-04	*CCNB1*, *CDC25C*, *CDC45*, *CDC6*, *FHL2*, *MCM10*, *PBK*, *TYMS*
Post-Translational Modification	6.72E-03- 1.25E-04	*CDC25C*, *CDC6*, *DIAPH3*
**Top Canonical Pathways**		
**Pathway**	**P-value**	**Selected Molecules**
Cell Cycle: G2/M DNA Damage Checkpoint Regulation	4.87E-04	*CCNB1*, *CDC25C*
Cell Cycle Control of Chromosomal Replication	6.36E-04	*CDC6*, *CDC45 *
Mitotic Roles of Polo-Like Kinase	8.05E-04	*CCNB1*, *CDC25C*
ATM Signaling	1.86E-03	*CCNB1*, *CDC25C*
dTMP De Novo Biosynthesis	3.37E-03	*TYMS*

This table contains the enrichment analysis results for genes associated with COVID-19 status. For the gene set enrichment analysis the IPA output contains the top five canonical pathways and top ten biological functions and disease associations.

**Table 2:** Regression models for genes associated with COVID-19 status.

Pathway		(Intercept)	PC1	PC2	Age	Gender (Male)	Charlson Score
**Cell Cycle: G2/M DNA Damage Checkpoint, Mitotic Roles of Polo-Like Kinase & ATM Signaling**	**Coef (SE)**	1.703 (1.791)	3.221 (0.687)	1.824 (1.292)	0.025 (0.036)	1.742 (0.937)	-0.091 (0.230)
	p-values *LRT	-	**<2e-16**	0.370	0.349	**0.047**	0.696
	**95% Conf.int**	(-1.711, 5.509)	(2.101, 4.870)	(-0.515, 4.595)	(-0.045, 0.098)	(0.030, 3.800)	(-0.529, 0.380)
	**%Var Explained**	-	**92.79%**	**7.21%**	-	-	-
**Cell Cycle Control of Chromosomal Replication**	**Coef (SE)**	5.645 (2.573)	-3.334 (0.861)	-3.781 (1.624)	-0.010 (0.042)	1.902 (1.012)	-0.179 (0.287)
	p-values *LRT	-	**<2e-16**	**0.022**	0.393	**0.043**	0.526
	**95% Conf.int**	(1.267, 11.687)	(-5.504, -2.015)	(-7.359, -0.694)	(-0.096, 0.073)	(0.080, 4.176)	(-0.789, 0.368)
	**%Var Explained**	-	**95.36%**	**4.64%**	-	-	-

Summary of multivariate logistic regression models with COVID-19 status as the outcome and the first two principal components used to summarize the enriched pathways associated with COVID-19 status as the predictors. The models are also adjusted for the clinical covariates: age, sex and Charlson comorbidity score. P-values for significance are determined via the likelihood ratio test (LRT).

**Fig 3:**
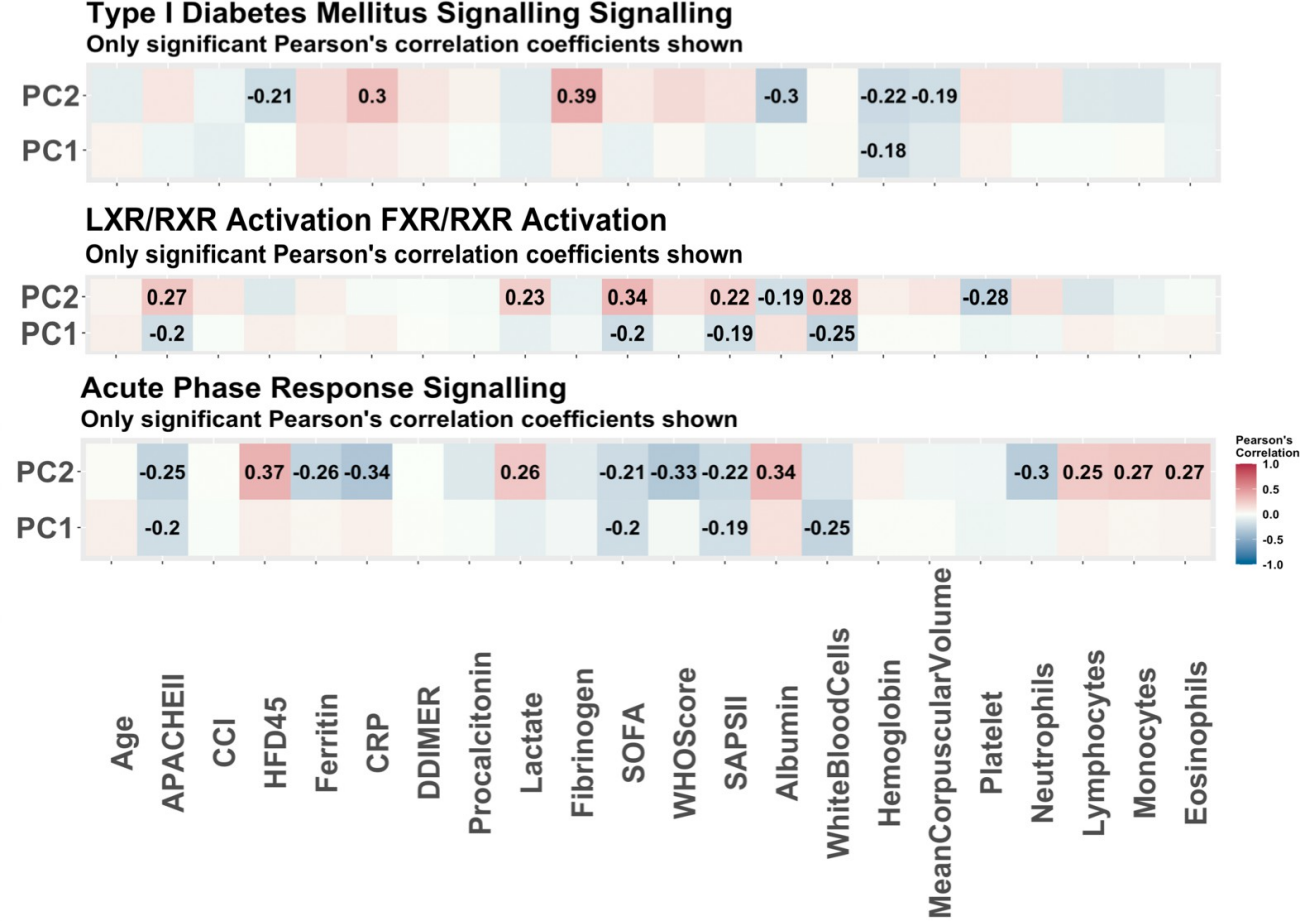
The Pearson correlations of principal components of pathways associated with disease status. The Pearson correlations of clinical variables and the principal components used to summarize the enriched pathways with COVID-19 status as predicted by IPA. These are the pathways predicted to be enriched based on the 22 proteins determined to be associated with COVID-19 status via stability selection. Only the correlations which were significant (p-value<0.05) are reported. The strongest correlations with the LXR/RXR activation and FXR/RXR activation pathways are with SOFA score and white blood cell count. The strongest correlation with the acute phase response signalling pathway is with HFD-45. With the diabetes mellitus signalling pathway, the top correlation is with fibrinogen.

**Fig 4:**
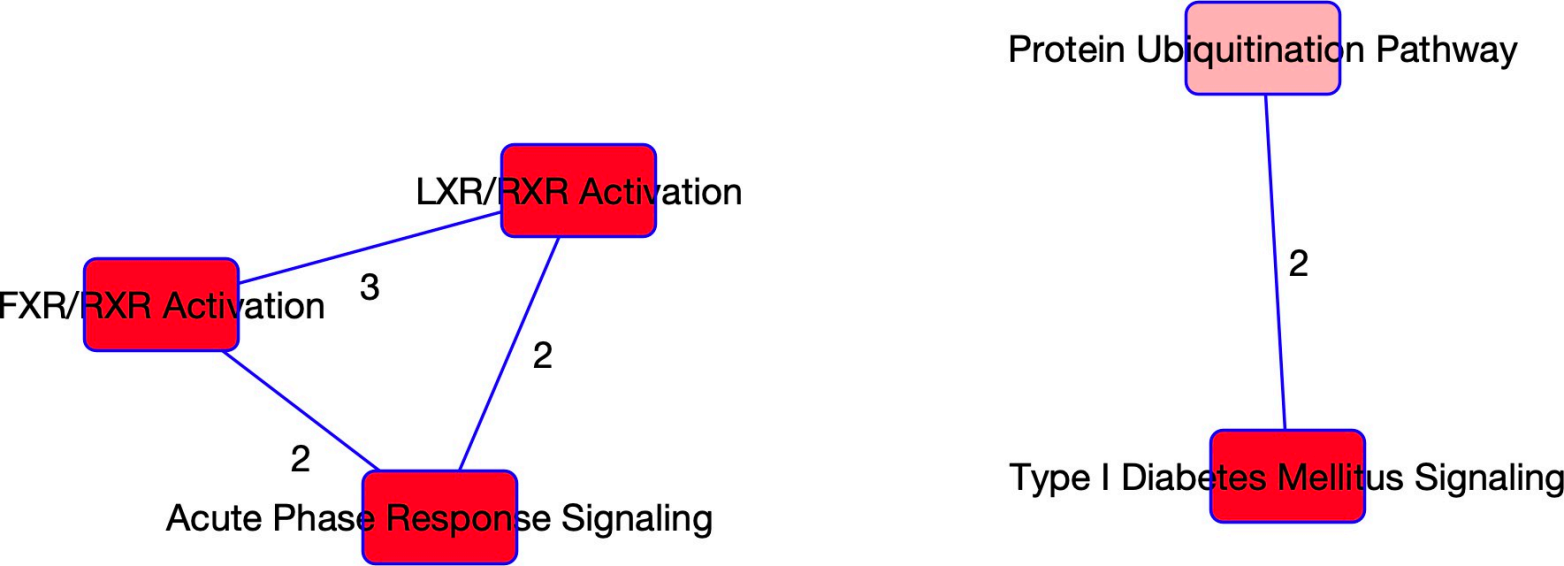
Overlapping networks associated with COVID-19. Visual of the overlapping networks enriched in COVID-19 as determined from the proteomics data. The nodes represent the networks, and the edges represent the overlapping proteins between the networks. The edge labels give the number of overlapping molecules between the networks.

The same procedure was applied to the proteomics data, and 22 molecules were determined to be associated with COVID-19 status (refer to S2 Table in S1 File). A summary of the enrichment analysis performed in IPA is available in Table 3. Of note, 9 of the molecules determined to be associated with COVID-19 status are suggested to be involved in neurological disease. These findings align with current studies which indicate that COVID-19 may be associated with certain neurological conditions such as ischemic strokes [15]. Further, a retrospective cohort study investigating psychiatric and neurological associations with COVID-19 diagnosis [16] found some evidence to show that incidences of multiple neurological conditions (e.g., strokes, anxiety) were higher in patients recovering from COVID-19 than the influenza. As in the case with the genes selected to be related to COVID-19 status (using RNAseq data), we observed a strong signature in the cell cycle. The top biological functions and diseases are also related to the cycle and inflammation and organismal injury. The top pathways enriched in the protein list are linked to the metabolic processes. For instance, the LXR/RXR activation pathway, which plays a key role in regulation of lipid metabolism, inflammation, and the cholesterol to bile acid catabolism process, was enriched [16]. The FXR/RXR activation pathway, which plays a role in the metabolic process and a moderator of bile, lipid and glucose homeostasis, was also enriched [17]. These findings are in agreement with the original study [2] which determined that a dysregulated lipid transport system is likely a key signature of COVID-19. The acute phase response signalling pathway which was also found to be significantly enriched plays a key role in the inflammatory response, which again indicates to us the unique immune response to COVID-19. Four of the top enriched pathways had more than one molecule selected so PCA was performed on the four pathways. The PC scores of the individuals relating to each pathway were further correlated with clinical covariates. A heat map of these correlations is provided in Fig 3. From the PCs of the pathways, some strong correlations with clinical covariates emerge. For instance, the strongest correlations in the LXR/RXR and FXR/RXR pathways, as well as the acute phase response signalling pathways are with the white blood cell count and albumin levels. This correlation is expected considering white blood cells and albumin are directly related to the inflammatory response [18]. In addition, logistic regression models were fitted using clinical covariates and the first two principal components from the pathways as predictors, and COVID-19 status as the outcome. The p-values associated with the predictors are reported in Table 4. As in the case with the RNAseq data, the PCs are statistically significant in the logistic regression models. Further, the Charlson comorbidity score is found to be significant in these models. A visual of the overlapping networks is available in Fig 4.

**Table 3:** Enrichment analysis of proteins associated with COVID-19 status.

Top Diseases and Biological Functions		
	**P-value range**	**Selected Molecules**
Neurological Disease	4.85E-02–1.25E-05	*C4A/C4B*, *CSF1R*, *HLA-B*, *HRG*, *HSPD1*, *LGALS3BP*, *LUM*, *PLTP*, *TTR*
Organismal Injury and Abnormalities	4.99E-02–1.25E-05	*APMAP*, *C4A/C4B*, *CKM*, *CRTAC1*, *CSF1R*, *HLA-B*, *HRG*, *HSPD1*, *IGHV1-2*, *LCP1*, *LGALS3BP*, *LUM*, *PLTP*, *SFTPB*, *TTR*
Metabolic Disease	2.15E-02–5.18E-05	*C4A/C4B*, *CKM*, *CSF1R*, *HRG*, *HSPD1*, *PLTP*, *SFTPB*, *TTR*
Psychological Disorders	3.78E-02–5.18E-05	*C4A/C4B*, *CSF1R*, *HLA-B*, *HRG*, *HSPD1*, *PLTP*, *TTR*
Inflammatory Response	4.50E-02–1.05E-04	*C4A/C4B*, *CKM*, *CSF1R*, *HLA-B*, *HRG*, *HSPD1*, *IGKV2-30*, *IGLV3-1*, *LCP1*, *LGALS3BP*, *PLTP*, *TTR*
Cellular Movement	3.75E-02–7.82E-06	*C4A/C4B*, *CSF1R*, *HRG*, *HSPD1*, *IGKV2-30*, *IGLV3-1*, *LCP1*, *LGALS3BP*
Protein Synthesis	3.22E-03–3.28E-05	*C4A/C4B*, *H3C15*, *HSPD1*, *SFTPB*, *TTR*
Cellular Compromise	1.38E-02–1.82E-04	*C4A/C4B*, *HLA-B*, *HRG*, *LGALS3BP*, *TTR*
Cell Death and Survival	4.75E-02- 2.62E-04	*C4A/C4B*, *CSF1R*, *HLA-B*, *HRG*, *HSPD1*, *SFTPB*
Cellular Development	4.75E-02–2.62E-04	*C4A/C4B*, *CSF1R*, *HLA-B*, *HSPD1*, *LUM*, *TTR*
**Top Canonical Pathways**		
**Pathway**	**P-value**	**Selected Molecules**
LXR/RXR Activation	1.48E-04	*C4A/C4B*, *PLTP*, *TTR*
FXR/RXR Activation	1.63E-04	*C4A/C4B*, *PLTP*, *TTR*
Acute Phase Response Signalling	4.67E-04	*C4A/C4B*, *HRG*, *TTR*
Type I Diabetes Mellitus Signaling	3.81E-03	*HLA-B*, *HSPD1*
Creatine-phosphate Biosynthesis	4.33E-03	*CKM*

This table contains the enrichment analysis results for proteins associated with COVID-19 status. For the protein set enrichment analysis the IPA output contains the top five canonical pathways and top ten biological functions and disease associations.

**Table 4:** Regression models for proteins associated with COVID-19 status.

Pathway		(intercept)	PC1	PC2	Age	Gender (Male)	Charlson Score
**LXR/RXR Activation FXR/RXR Activation**	**Coef (SE)**	36.911 (6.645)	-3.915 (2.181)	5.363 (2.418)	-0.135 (0.132)	-2.315 (3.225)	-1.561 (0.836)
	p-values *LRT	-	**0.003**	**0.010**	**0.002**	0.741	0.062
	**95% Conf.int**	(23.886, 49.936)	(-8.190, 0.361)	(0.623, 10.102)	(-0.393, 0.123)	(-8.636, 4.005)	(-3.199, 0.076)
	**%Var Explained**	-	**40.97%**	**34.26%**	-	-	-
**Acute Phase Response Signalling**	**Coef (SE)**	38.192 (6.196)	2.337 (0.849)	3.028 (0.903)	-0.106 (0.122)	-1.899 (3.041)	-2.446 (0.818)
	p-values *LRT	**-**	**0.029**	**0.001**	**<2e-16**	0.921	**0.003**
	**95% Conf.int**	(26.048, 50.336)	(0.674, 4.000)	(1.259, 4.797)	(-0.346, 0.134)	(-7.860, 4.062)	(-4.049, -0.843)
	**%Var Explained**	-	**49.99%**	**33.18%**	-	-	-
**Type I Diabetes Mellitus Signaling**	**Coef (SE)**	2.358(1.358)	-0.938 (0.198)	1.179 (0.369)	0.008 (0.025)	0.706 (0.654)	-0.163 (0.154)
	p-values *LRT	-	**<2e-16**	**<2e-16**	0.976	0.274	0.291
	**95% Conf.int**	(-0.162, 5.243)	(-1.373, -0.586)	(0.547, 2.008)	(-0.043, 0.058)	(-0.565, 2.041)	(-0.471, 0.144)
	**%Var Explained**	-	**61.52%**	**38.48%**	-	-	-

Summary of multivariate logistic regression models with COVID-19 status as the outcome and the principal components used to summarize the enriched pathways associated with COVID-19 status as the predictors. The models are also adjusted for the clinical covariates age, sex and Charlson comorbidity score. P-values for significance are determined via the likelihood ratio test (LRT).

When stability selection was used to select metabolites associated with COVID-19 status, 25 molecules were selected, however only 11 of the molecules were able to be mapped to known pathways. A summary of the enrichment analysis performed on these molecules in IPA is provided in Table 5. Similar to the RNAseq and proteomics analyses we notice that one of the main effects of COVID-19 seems to be on the cell cycle. We observe molecules that are related to infectious disease and antimicrobial response involved in the signature of COVID-19. In addition, two molecules which were selected are related to neurological disease which is in agreement with findings from the proteomics data. As none of the top pathways contain more than one molecule, PCA was not used to further look into these pathways. Notice that most of the selected pathways are centred around myo-inositol which plays a role in pathways that synthesize vitamin C. Further analysis into these pathways could reveal if vitamin C is a potential treatment for COVID-19.

**Table 5:** Enrichment analysis of metabolites associated with COVID-19 status.

Top Diseases and Biological Functions		
	**P-value range**	**Selected Molecules**
Antimicrobial Response	1.22E-04–1.22E-04	*Sucrose*
Dermatological Diseases and Conditions	1.22E-04–1.22E-04	*Sucrose*
Herditary Disorder	7.32E-04–1.22E-04	*Myo-inositol*
Infectious Diseases	9.12E-03–1.22E-04	*Sucrose*
Neurological Disease	7.91E-03–1.22E-04	*Myo-inositol*, *sucrose*
Small Molecule Biochemistry	4.56E-02–1.22E-04	*Sucrose*, *L-kynurenine*
Cell Cycle	2.44E-04–2.44E-04	*L-kynurenine*
Cell Morphology	2.44E-04–2.44E-04	*Sucrose*
Cellular Compromise	1.73E-02–2.44E-04	*Sucrose*, *L-kynurenine*
Cellular Assembly and Organization	4.72E-02–3.66E-04	*Myo-inositol*, *sucrose*
**Top Canonical Pathways**		
**Pathway**	**P-value**	**Selected Molecules**
Myo-inositol Biosynthesis	1.10E-03	*Myo-inositol*
Sucrose Degradation V	2.32E-03	*Sucrose*
D-myo inositol(1,4,5)-triphosphate Degradation	2.68E-03	*Myo-inositol*
Superpathway of D-myo inositol(1,4,5)-triphosphate Metabolism	3.90E-03	*Myo-inositol*
D-myo inositol(1,4,5)-triphosphate Biosynthesis	4.26E-03	*Myo-inositol*

This table contains the enrichment analysis results for metabolites associated with COVID-19 status. For the metabolite set enrichment analysis the IPA output contains the top five canonical pathways and top ten biological functions and disease associations.

**Fig 5:**
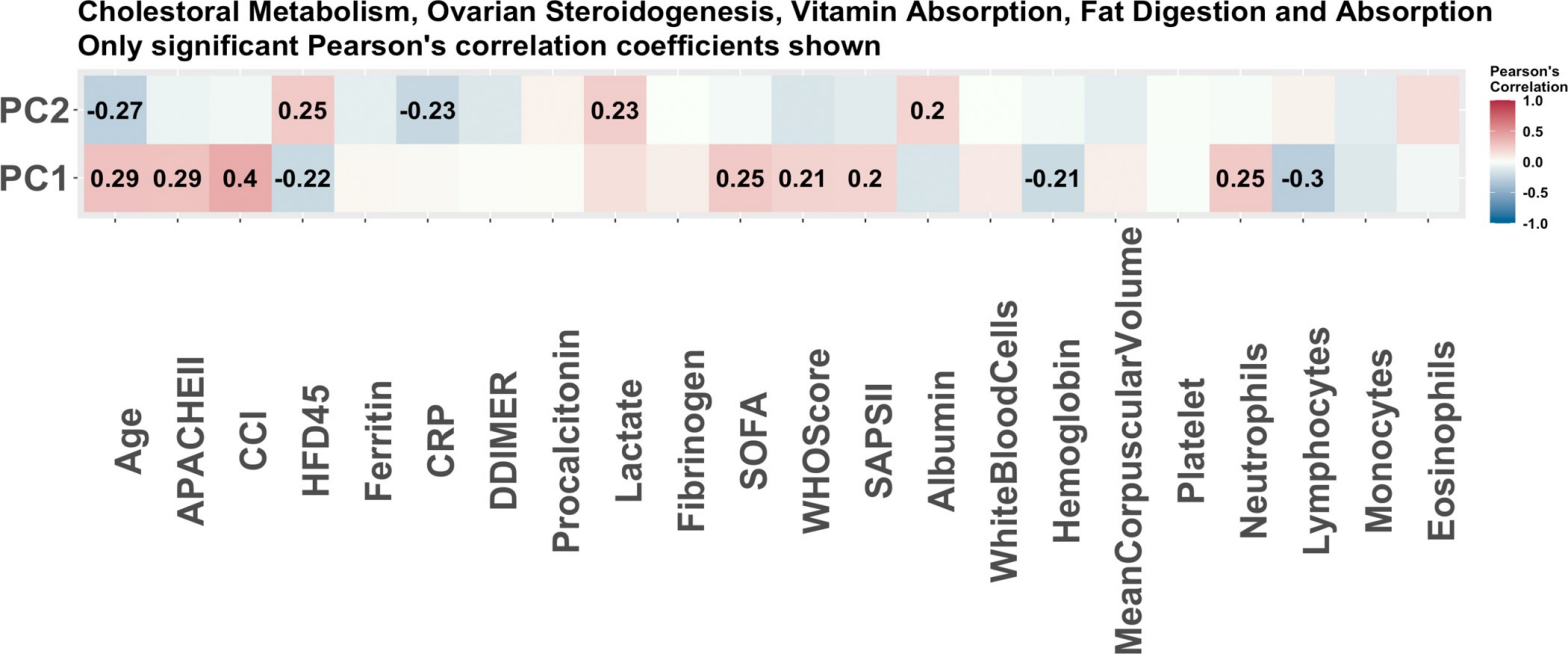
The Pearson correlations of principal components of pathways associated with disease status. The Pearson correlations of clinical covariates and the principal components used to summarize the enriched pathways with COVID-19 status as predicted by LIPEA. These are the pathways predicted to be enriched based on the 9 lipids determined to be associated with COVID-19 status via stability selection. Only the correlations which were significant (p-value<0.05) are reported. The strongest correlation is with the Charlson score.

From the lipidomics data, 12 molecules were selected to be associated with COVID-19 status via stability selection, however only 9 of these were annotated and input into LIPEA software [19] for enrichment analysis. Ten (10) pathways were determined to be enriched. The summary of the top five pathways is available in Table 6. The pathways which have more than one molecule selected and a p-value less than 0.05 were further analyzed through their principal components. The same molecules were selected for four statistically significant pathways (based on unadjusted p-values). These pathways include cholesterol metabolism, fat digestion and absorption, vitamin digestion and absorption, and ovarian steroidogenesis. This results in one correlation matrix with clinical covariates and PCs as shown in Fig 5, as well as one regression model with COVID-19 status as the response. The cholesterol metabolism pathway was enriched and this aligns with other research papers which show a unique effect of COVID-19 on cholesterol metabolism [20]. The fact that much of the molecules selected are related to ovarian steroidogenesis is in agreement with current studies that ovarian injury and reproductive endocrine disorder can be observed in women with COVID-19 [21]. A summary of the regression model is available in Table 7. It shows that the first PC scores are significant in the model. The lipidomics analysis gives us similar results to the proteomics data, as the significant pathways tend to be related to metabolic processes. The top correlations with the principal components are found to be the Charlson comorbidity score, and both the SOFA and APACHE II scores (see Fig 5).

**Table 6:** Enrichment analysis of lipids associated with COVID-19 status.

Enriched Pathways				
Pathway	p-value	Benjamini correction	Bonferroni correction	Selected Molecules
**Fat digestion and absorption**	0.006718104	0.036949572	0.073899145	*ST32*:*5;O10*, *ST24*:*4;O6*
**Cholesterol metabolism**	0.006718104	0.036949572	0.073899145	*ST32*:*5;O10*, *ST24*:*4;O6*
**Basal cell carcinoma**	0.016853933	0.061797753	0.185393258	*ST32*:*5;O10*
**Vitamin digestion and absorption**	0.023682416	0.065126643	0.260506571	*ST32*:*5;O10*, *ST24*:*4;O6*
**Ovarian steroidogenesis**	0.033605179	0.073931393	0.369656964	*ST32*:*5;O10*, *ST24*:*4;O6*

This table contains the enrichment analysis results for lipids associated with COVID-19 status. For the lipidomics set enrichment analysis from LIPEA the top 10 enriched pathways are summarized.

**Table 7:** Regression models for proteins associated with COVID-19 status.

Pathway		(intercept)	PC1	PC2	Age	Gender (Male)	Charlson Score
**Cholesterol metabolism, Ovarian steroidogenesis, Vitamin digestion and absorption, Fat digestion and absorption**	**Coef (SE)**	-3.200 (1.208)	-0.315 (0.104)	-0.346 (0.116)	0.023 (0.021)	-0.494 (0.519)	0.058 (0.142)
	p-values *LRT	-	**0.001**	**0.001**	0.518	0.279	0.111
	**95% Conf.int**		(-0.537, -0.124)	(-0.588, -0.129)	(-0.019, 0.066)	(-1.530, 0.526)	(-0.227, 0.331)
	**%Var Explained**	-	**51.77%**	**36.71%**	-	-	-

Summary of multivariate logistic regression models with COVID-19 status as the outcome and the principal components used to summarize the enriched pathways associated with COVID-19 status as the predictors. The models are also adjusted for the clinical covariates age, sex and Charlson comorbidity score. P-values for significance are determined via the likelihood ratio test (LRT).

### 3.2 COVID-19 severity (all patients)

When stability selection was employed on the RNAseq data for all patients (i.e. patients with/without COVID-19), with severity (measured as hospital free days) as the outcome, 25 genes were selected. None of these selected genes were previously selected in relation to disease status. These genes again were processed in IPA software and the results are summarized similarly in Table 8. All of the pathways selected had only one molecule so further analysis into the pathways was not done. Though no PCA was performed on these pathways, there are some interesting points to note. For one, we again observe that the cell cycle is one of the most significant bio functions. We also observe that the airway inflammation in asthma pathway is enriched which aligns with current studies that show that asthma plays a significant role in respiratory disease and COVID-19 outcomes. The other top pathways that were selected based on the RNAseq data are related to inflammatory response and metabolic processes. Specifically, we find the enriched pathways centered around vitamin A metabolism. The retinoate biosynthesis pathways as well as the visual cycle are all centered around the metabolism of vitamin A. Vitamin A plays many important roles in human biology but some of the most important roles are found in human vision and bone fragility/formation. We find the focus on vitamin A interesting as recently vitamin A has been explored as an option to treat COVID-19. Further investigation into vitamin A regulating pathways could provide more insight into the potential efficacy of the treatment [22]. We also find that 23 of the molecules selected are determined to be associated with cancer. This is further evidence to support current studies which state that individuals with cancer or recently recovered from cancer are at higher risk of severe outcomes due to a weakened immune system [23, 24].

**Table 8:** Enrichment analysis of genes associated with COVID-19 severity (all patients).

Top Diseases and Biological Functions		
	**P-value range**	**Selected Molecules**
Cancer	4.98E-02–1.11E-03	*C17orf97*, *CNR1*, *CXXC4*, *DNAAF1*, *EPAS1*, *GOLGA8T*, *HEPHL1*, *LRGUK*, *MEIS3*, *MFAP4*, *MYO5B*, *NECAB2*, *NFIB*, *OR52N4*, *PRSS50*, *RBP5*, *RIPK4*, *RNASE2*, *SEZ6L*, *SPATA20*, *TMEM52B*, *UGT2B11*, *ZNF221*
Connective Tissue Disorders	4.47E-02–1.11E-03	*ESPAS1*, *NFIB*, *HEPHL*, *MFAP4*, *CNR1*
Dermatological Diseases and Conditions	1.11E-03–1.11E-03	*RIPK4*, *HEPHL1*
Developmental Disorder	4.23E-02–1.11E-03	*EPAS1*, *RIPK4*, *NFIB*, *HEPHL1*, *MYO5B*, *DNAAF1*, *MFAP4*, *CNR1*
Endocrine System Disorders	1.10E-02–1.11E-03	*EPAS1*, *CNR1*
Cellular Movement	3.16E-02–1.11E-03	*CNR1*, *DNAAF1*
Cellular Development	4.78E-02–5.33E-03	*CXXC4*, *EPAS1*, *RNASE2*, *RIPK4*, *NFIB*
Cellular Growth and Proliferation	4.78E-02–5.33E-03	*CXXC4*, *EPAS1*, *RIPK4*, *NFIB*
Cell Death and Survival	6.63E-03–6.63E-03	*RNASE2*
Amino Acid Metabolism	1.54E-02–7.73E-03	*EPAS1*, *CNR1*
**Top Canonical Pathways**		
**Pathway**	**P-value**	**Selected Molecules**
Retinoate Biosynthesis II	4.42E-03	*RBP5*
The Visual Cycle	2.08E-02	*RBP5*
Thyroid Hormone Metabolism II	3.38E-02	*UGT2B11*
Airway Inflammation in Asthma	3.49E-02	*RNASE2*
Retinoate Biosynthesis I	3.49E-02	*RBP5*

This table contains the enrichment analysis results for genes associated with disease severity when using all patients. For the gene set enrichment analysis the IPA output contains the top five canonical pathways and top ten biological functions and disease associations.

**Fig 6:**
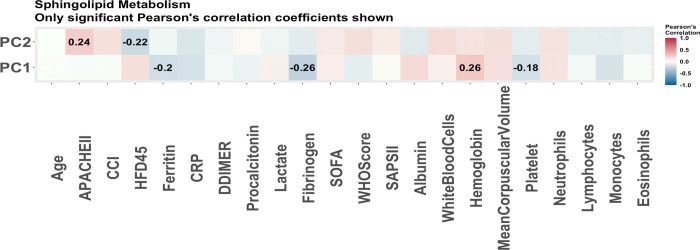
The Pearson correlations of principal components of pathways associated with disease severity (all patients). The Pearson correlations of clinical covariates and the principal components used to summarize the enriched pathways with disease severity as predicted by IPA. These are the pathways predicted to be enriched based on the 67 proteins determined to be associated with disease severity via stability selection. Only the correlations which were significant (p-value<0.05) are reported. These pathways all have multiple significant correlations with clinical covariates.

**Fig 7:**
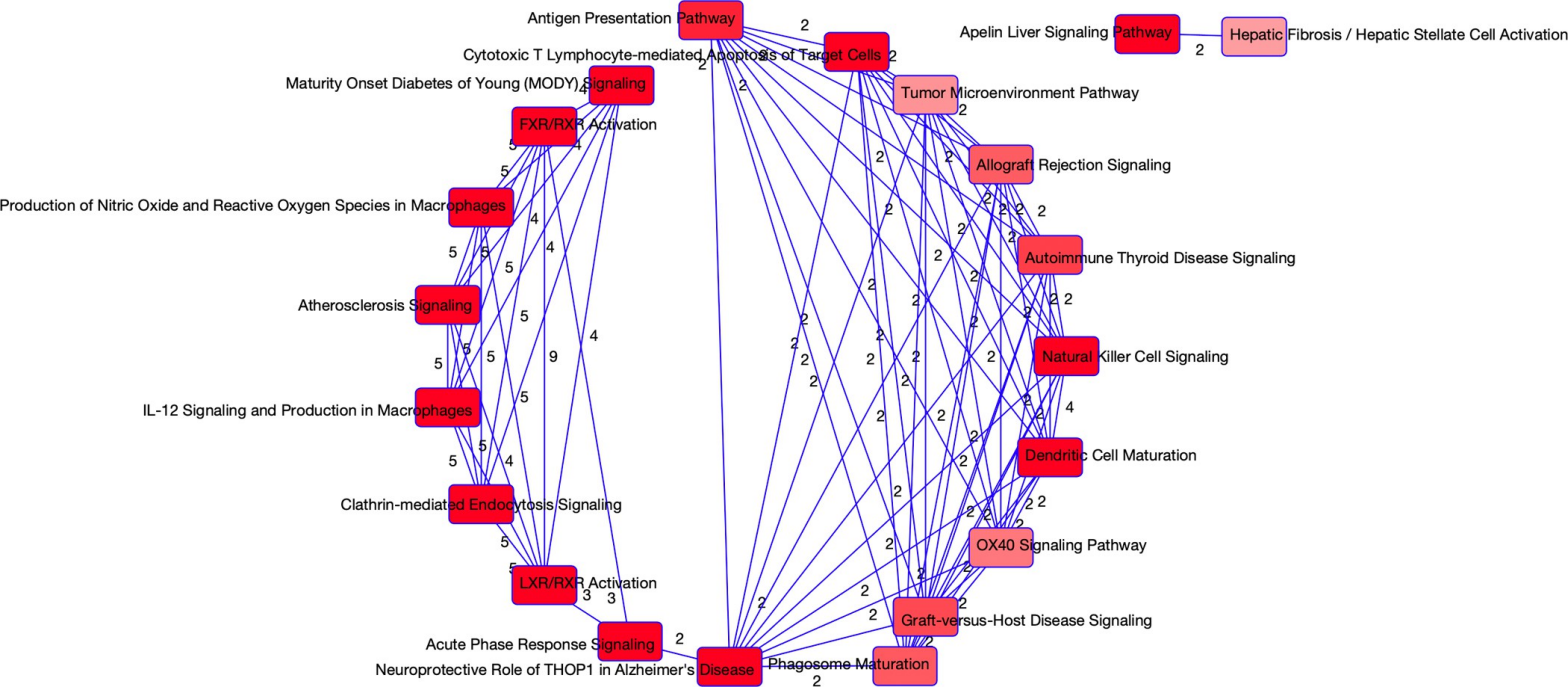
Overlapping networks associated with COVID-19 visual of the overlapping networks enriched in COVID-19 as determined from the proteomics data. The nodes represent the networks and the edges represent the overlapping proteins between the networks. The edge labels give the number of overlapping molecules between the networks.

From the stability selection of proteins for HFD-45 using all patients, we ended up with 69 molecules which were put into IPA for enrichment analysis. Of these molecules, there were 2 which were unable to be mapped to any pathways. None of the 69 proteins were selected in association with COVID-19 status. The summary of the IPA outputs is available in Table 9 and the pathways with more than one molecule are further analyzed through PCA. We observed similar results in the significant bio functions as was indicated in the RNAseq data, with the cell cycle being one of the significant mechanisms. There were also 38 molecules associated with inflammatory response that are related to disease severity, which emphasizes the unique immune response that plays a significant role in clinical outcomes. From the proteomics data the enriched pathways are once again determined to be the FXR/RXR and LXR/RXR pathways, and the acute phase response signalling pathway. These were again found to be significant in the regression models with HFD as the outcome, summarized in Table 10. We also find that the atherosclerosis signalling pathway is enriched which again is closely related to metabolic processes especially cholesterol metabolism. It has been shown that COVID-19 may magnify the evolution of atherosclerosis, which is a disease when plaque builds up in the arteries [25]. The principal components from this pathway were also found to be significant in the regression model, and had a strong correlation with SOFA score and lymphocyte volumes as displayed in the correlation heatmaps for the PCs found in Fig 6. The final enriched pathway was the neuroprotective role of THOP1 in Alzheimer’s disease; this pathway has a strong correlation with lymphocyte levels and neutrophil percent. This relationship with Alzheimer’s has been analyzed in other studies which explore the long term effects of COVID-19 [26]. The PCs of this pathway were also significant in the disease severity regression model. Again a visual of the overlapping networks is provided in Fig 7.

**Table 9:** Enrichment analysis of proteins associated with COVID-19 severity (all patients).

Top Diseases and Biological Functions		
	**P-value range**	**Selected Molecules**
Inflammatory Response	2.10E-02–4.69E-13	*AGT*, *APOA2*, *ANPEP*, *APOM*, *ACSL6*, *B4GALT1*, *C4A/C4B*, *CHI3L1*, *CFHR1*, *CTSD*, *CD5L*, *FCGR3A/FCGR3B*, *HBA1/HBA2*, *HBB*, *HLA-A*, *HLA-C*, *HP*, *HRG*, *ITIH3*, *IGKV1D-8*, *JCHAIN*, *LTA4H*, *LCP1*, *MRC1*, *PPBP*, *PSMA5*, *SERPINA3*, *SPP2*, *TIMP1*, *TNC*, *TNXB*, *THBS4*, *VNN1*, *IGHV1OR15-1*, *IGHV2-70*, *IGLC7*, *IGLV1-44*, *MASP1*
Infectious Diseases	1.51E-02–6.74E-12	*ANPEP*, *AGT*, *APOA2*, *APOD*, *APOM*, *B4GALT1*, *C4A/C4B*, *FCGR3A/FCGR3B*, *H2BC15*, *HBA1/HBA2*, *HBB*, *HLA-A*, *HLA-C*, *HP*, *HRG*, *ITIH3*, *LTA4H*, *MRC1*, *PCYOX1*, *PPBP*, *PSMA5*, *SERPINA3*, *SFTPB*, *TIMP1*, *VNN1*
Endocrine System Disorders	3.47E-03–7.74E-09	*AGT*, *APOD*, *APOM*, *C4A/C4B*, *CETP*, *CHI3L1*, *COL18A1*, *CTSD*, *FBLN5*, *FCGR3A/FCGR3B*, *HBA1/HBA2*, *HBB*, *HLA-A*, *HLA-C*, *HP*, *IGFBP7*, *TNC*, *TNXB*
Gastrointestinal Disease	2.10E-02–7.74E-09	*AGT*, *APOA2*, *APOD*, *APOF*, *APOM*, *C4A/C4B*, *CD5L*, *CETP*, *CHI3L1*, *CNDP1*, *COL18A1*, *CTSD*, *FBLN5*, *FCGR3A/FCGR3B*, *HBA1/HBA2*, *HBB*, *HLA-A*, *HLA-C*, *HP*, *IGFBP7*, *JCHAIN*, *MRC1*, *PROZ TIMP1*, *TNC*, *TNXB*
Metabolic Disease	9.07E-03–7.74E-09	*ANGPTL3*, *AGT*, *APOA2*, *APOC4-APOC2*, *APOD*, *APOM*, *B4GALT1*, *C4A/C4B*, *CETP*, *CHI3L1*, *CHL1*, *COL18A1*, *CTSD*, *FBLN5*, *FCGR3A/FCGR3B*, *GNPTG*, *HBA1/HBA2*, *HBB*, *HLA-A*, *HLA-C*, *HP*, *HRG*, *IGFBP7*, *MRC1*, *PROZ*, *SERPINA3*, *SFTPB*, *TIMP1*, *TNC*, *TNXB*
Cellular Compromise	1.21E-02–4.69E-13	*ANPEP*, *B4GALT1*, *C4A/C4B*, *CHI3L1*, *CTSD*, *FCGR3A/FCGR3B*, *HBB*, *HLA-C*, *HP*, *HRG*, *ITIH3*, *JCHAIN*, *LTA4H*, *PPBP*, *PSMA5*, *SERPINA3*, *SPP2*, *TIMP1*, *VNN1*
Cellular Movement	2.10E-02–2.32E-10	*AGT*, *ANGPTL3*, *ANPEP*, *APOD*, *C4A/C4B*, *CFHR1*, *CHI3L1*, *CHL1*, *COL18A1*, *CTSD*, *FBLN5*, *HLA-A*, *HRG*, *IGFBP7*, *IGHV1OR15-1*, *IGHV2-70*, *IGLC7*, *IGLV1-44*, *JCHAIN*, *LCP1*, *PPBP*, *SERPINA3*, *SPARCL1*, *THBS4*, *TIMP1*, *TNC*, *WARS1*
Cellular Function and Maintenance	1.86E-02–1.47E-09	*ANK1*, *ANPEP*, *APOD*, *CD5L*, *COL18A1*, *FCGR3A/FCGR3B*, *HBA1/HBA2*, *HBB*, *HP*, *HRG*, *IGHV1OR15-1*, *IGHV2-70*, *IGLC7*, *IGLV1-44*, *JCHAIN*, *LCP1*, *MASP1*, *MRC1*, *TIMP1*
Protein Synthesis	6.06E-03–1.97E-08	*AGT*, *APOA2*, *C4A/C4B*, *CCT6A*, *CNDP1*, *CTSD*, *IGFBP7*, *LTA4H*, *PCYOX1*, *SFTPB*, *SPARCL1*, *SPP2*, *TIMP1*, *TNC*, *WARS1*
Cell Death and Survival	1.81E-02–1.62E-06	*ANK1*, *ANPEP*, *APOD*, *C4A/C4B*, *CHI3L1*, *CFHR1*, *COL18A1*, *CTSD*, *FCGR3A/FCGR3B*, *HBA1/HBA2*, *HBB*, *HRG*, *PPBP*, *PSMA5*, *SERPINA3*, *SFTPB*, *TIMP1*, *TNC*
**Top Canonical Pathways**		
**Pathway**	**P-value**	**Selected Molecules**
LXR/RXR Activation	1.04E-10	*AGT*, *APOA2*, *APOD*, *APOF*, *APOM*, *C4A/C4B*, *CETP*, *PCYOX1*, *PON3*
FXR/RXR Activation	1.39E-10	*AGT*, *APOA2*, *APOD*, *APOF*, *APOM*, *C4A/C4B*, *CETP*, *PCYOX1*, *PON3*
Acute Phase Response Signalling	1.16E-06	*AGT*, *APOA2*, *C4A/C4B*, *HP*, *HRG*, *ITIH3*, *SERPINA3*
Atherosclerosis Signalling	1.97E-06	*APOA2*, *SPOD*, *APOF*, *APOM*, *COL18A1*, *PCYOX1*
Neuroprotective Role of THOP1 in Alzheimers Disease	2.00E-05	*AGT*, *HLA-A*, *HLA-C*, *MASP1*, *SERPINA3*

This table contains the enrichment analysis results for proteins associated with disease severity when using all patients. For the protein set enrichment analysis the IPA output contains the top five canonical pathways and top ten biological functions and disease associations.

**Table 10:** Regression models for proteins associated with COVID-19 severity (all patients).

Pathway		(Intercept)	PC1	PC2	Age	Gender (Male)	Charlson Score
**LXR/RXR Activation/FXR/RXR Activation**	**Coef (SE)**	33.585 (5.321)	-4.715 (0.737)	2.468 (0.941)	-0.066 (0.102)	-3.022 (2.544)	-1.088 (0.661)
	p-values *LRT	-	**<2e-16**	**0.002**	**0.024**	0.328	0.100
	**95% Conf.int**	(23.157, 44.013)	(-6.159, -3.271)	(0.623, 4.314)	(-0.265, 0.134)	(-8.008, 1.963)	(-2.383, 0.207)
	**%Var Explained**	-	**37.37%**	**22.96%**	-	-	-
**Acute Phase Response Signalling**	**Coef (SE)**	33.709 (4.689)	-7.417 (0.883)	4.620 (0.976)	-0.041 (0.090)	-4.843 (2.249)	-1.258 (0.583)
	p-values *LRT	-	**<2e-16**	**<2e-16**	**0.010**	0.058	**0.031**
	**95% Conf.int**	(24.519, 42.899)	(-9.147, -5.687)	(2.708, 6.533)	(-0.218, 0.136)	(-9.251, -0.436)	(-2.401, -0.114)
	**%Var Explained**	-	**35.29%**	**27.82%**	-	-	-
**Atherosclerosis Signalling **	**Coef (SE)**	37.090 (5.250)	-4.756 (0.671)	-5.971 (1.469)	-0.116 (0.103)	-3.809 (2.569)	-1.208 (0.677)
	p-values *LRT	**-**	**<2e-16**	**<2e-16**	**0.002**	0.245	0.075
	**95% Conf.int**	(26.801, 47.379)	(-6.072, -3.441)	(-8.850, -3.092)	(-0.319, 0.086)	(-8.844, 1.226)	(-2.535, 0.119)
	**%Var Explained**	-	**64.54%**	**16.81%**	-	-	-
**Neuroprotective Role of THOP1 in Alzheimers Disease**	**Coef (SE)**	42.017 (6.043)	2.078 (0.795)	-0.012 (0.905)	-0.184 (0.117)	-5.208 (2.981)	-1.049 (0.772)
	p-values *LRT	-	**0.011**	0.956	**0.001**	0.103	0.174
	**95% Conf.int**	(30.172, 53.862)	(0.521, 3.636)	(-1.786, 1.762)	(-0.413, 0.045)	(-11.051, 0.635)	(-2.561, 0.464)
	**%Var Explained**	-	**46.06%**	**32.61%**	-	-	-

Summary of multivariate linear regression models with COVID-19 severity as the outcome using all patients, and the principal components used to summarize the enriched pathways associated with disease severity status as the predictors. The models are also adjusted for the clinical covariates age, sex and Charlson comorbidity score. P-values for significance are determined via the likelihood ratio test (LRT).

Stability selection of the metabolomics data resulted in 41 selected molecules with respect to disease severity as measured by HFD-45. Twenty of these molecules were found to be associated with COVID-19 status as well. Of the 41 molecules, 18 were able to be mapped to known pathways and hence were analyzed. Because there is significant overlap with the molecules selected with respect to COVID-19 status we observe similar results here as summarized in Table 11.

**Table 11:** Enrichment analysis of metabolites associated with COVID-19 severity (all patients).

Top Diseases and Biological Functions		
	**P-value range**	**Selected Molecules**
Cardiovascular Disease	2.23E-02–5.73E-04	*Myo-inositol*, *sucrose*, *uric acid*
Endocrine System Disorders	3.99E-02–5.73E-04	*Myo-inositol*, *sucrose*, *uric acid*
Hemtological Disease	3.11E-02–5.73E-04	*Myo-inositol*, *sucrose*, *uric acid*
Metabolic Disease	3.11E-02–5.73E-04	*Myo-inositol*, *salicylic acid*, *sucrose*, *uric acid*
Organismal Injury and Abnormalities	4.64E-02–5.73E-04	*L-kynurenine*, *myo-inositol*, *salicylic acid*, *sucrose*, *uric acid*
Cell Death and Survival	3.99E-02–4.50E-04	*L-kynurenine*, *salicylic acid*, *sucrose*, *uric acid*
Cellular Compromise	2.67E-02–4.50E-04	*L-kynurenine*, *sucrose*, *uric acid*
Carbohydrate Metabolism	3.33E-02–7.87E-04	*Myo-inositol*, *salicylic acid*, *sucrose*, *uric acid*
Cell-To-Cell Signaling and Interaction	4.86E-02–2.20E-03	*L-kynurenine*, *sucrose*, *uric acid*
Cell Morphology	2.25E-03–2.25E-03	*Sucrose*
**Top Canonical Pathways**		
**Pathway**	**P-value**	**Selected Molecules**
Myo-inositol Biosynthesis	8.97E-03	*Myo-inositol*
D-myo inositol(1,4,5)-triphosphate Degradation	1.12E-02	*Myo-inositol*
Urate Biosynthesis/Inosine 5’-phosphate Degradation	2.01E-02	*Uric acid*
Superpathway of D-myo inositol(1,4,5)-triphosphate Metabolism	2.01E-02	*Myo-inositol*
Sucrose Degradation V	2.23E-02	*Sucrose*

This table contains the enrichment analysis results for metabolites associated with disease severity when using all patients. For the metabolite set enrichment analysis the IPA output contains the top five canonical pathways and top ten biological functions and disease associations.

**Fig 8:**
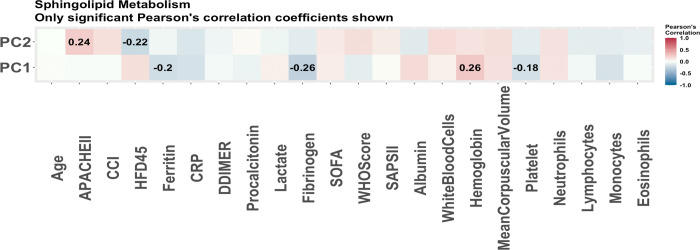
The Pearson correlations of principal components of pathways associated with disease severity (all patients). The Pearson correlations of clinical covariates and the principal components used to summarize the enriched pathways with disease severity using all patients as predicted by LIPEA. These are the pathways predicted to be enriched based on the 11 lipids determined to be associated with disease severity via stability selection which were able to be mapped to known pathways. Only the correlations which were significant (p-value<0.05) are reported. The strongest correlations are with fibrinogen and hemoglobin.

From the lipidomics data, 53 lipids were selected via stability selection with HFD-45 as the outcome. Only 36 of the molecules were annotated and hence were put into the LIPEA software. Of these 36 molecules, only 11 were able to be mapped to pathways. The top five enriched pathways are presented in Table 12. The sphingolipid metabolism pathway was selected based on more than one molecule hence it is further analyzed via principal components analysis. The first PC is found to be significant in the linear regression model summarized in Table 13. The first PC also has a strong correlation with hemoglobin levels and fibrinogen levels as shown in Fig 8. It appears this pathway displays less significance in disease outcome than the pathways enriched in the previous omics data. The sphingolipid metabolism is particularly interesting as this class of lipids is known for participation in the immune system and current studies are looking at these lipids as a possible treatment for COVID-19 [27]. Another point of interest is that cholesterol esters are selected as driving molecules of clinical outcomes. In another study analysing COVID-19 relationship with lipophagy in Vero E6 cells, these lipids were found to be significantly decreased in COVID-19 [28].

**Table 12:** Enrichment analysis of lipids associated with COVID-19 severity (all patients).

Enriched Pathways				
Pathway	p-value	Benjamini correction	Bonferroni correction	Selected Molecules
**Sphingolipid metabolism**	6.94137E-06	0.000104121	0.000104121	*HexCer36*:*2;O2*, *HexCer40*:*2;O2*, *HexCer44*:*0;O2*, *SPBP18*:*1;O2 *
**Fat digestion and absorption**	0.018723372	0.084269663	0.280850581	*CE*.*18*.*0*
**Cholesterol metabolism**	0.018723372	0.084269663	0.280850581	*CE*.*18*.*0*
**Sphingolipid signaling pathway**	0.023682416	0.084269663	0.355236233	*SPBP18*:*1;O2*
**Basal cell carcinoma**	0.028089888	0.084269663	0.421348315	*CE*.*18*.*0*

This table contains the enrichment analysis results for lipids associated with disease severity when using all patients. For the lipidomics set enrichment analysis from LIPEA the top 10 enriched pathways are summarized.

**Table 13:** Regression models for lipids associated with COVID-19 severity (all patients).

Pathway		(Intercept)	PC1	PC2	Age	Gender (Male)	Charlson Score
**Sphingolipid metabolism**	**Coef (SE)**	38.733 (6.040)	1.2801 (0.580)	-0.648 (0.758)	-0.144 (0.116)	-2.279 (3.01)	-1.318 (0.768)
	p-values *LRT	-	**0.031**	0.394	**0.001**	0.310	0.061
	**95% Conf.int**	(26.772,50.694)	(0.130, 2.430)	(-2.150, 0.853)	(-0.374, 0.087)	(-8.234, 3.676)	(-2.840, 0.203)
	**%Var Explained**	-	**39.35%**	**23.08%**	-	-	-

Summary of multivariate linear regression models with COVID-19 severity as the outcome using all patients, and the principal components used to summarize the enriched pathways associated with disease severity as the predictors. The models are also adjusted for the clinical covariates age, sex and Charlson comorbidity score. P-values for significance are determined via the likelihood ratio test (LRT).

### 3.3 COVID-19 severity (COVID-19 patients only)

**Fig 9:**
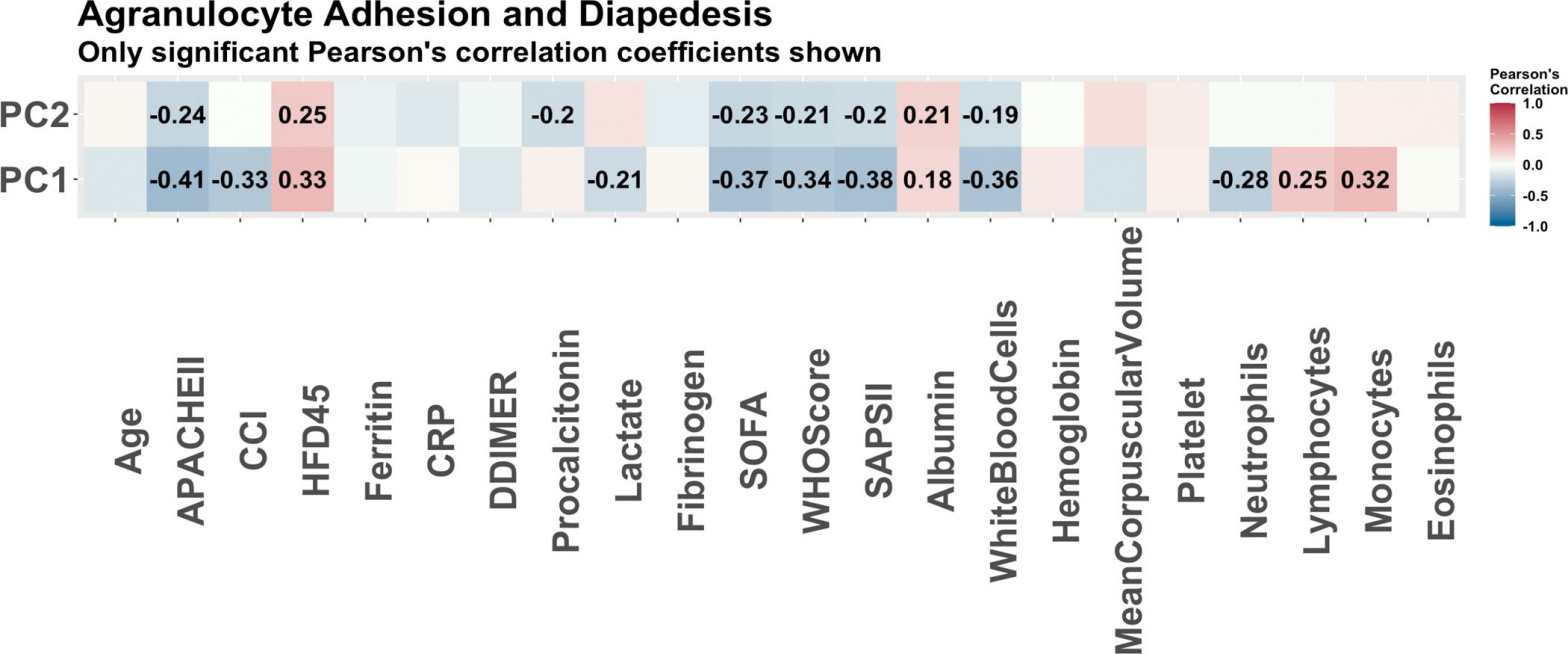
The Pearson correlations of principal components of pathways associated with disease severity (COVID-19 patients). The Pearson correlations of clinical covariates and the principal components used to summarize the enriched pathways with COVID-19 severity as predicted by IPA. These are the pathways predicted to be enriched based on 17 genes determined to be associated with COVID-19 severity via stability selection. Only the correlations which were significant (p-value<0.05) are reported. This pathway has many significant correlations with clinical covariates, especially measures of disease severity.

Stability selection of genes significant in predicting HFD-45 from the 99 patients with COVID-19 resulted in 17 selected genes. These genes were input into the IPA software and again the outputs are summarized in Table 14. Of the 17 molecules selected via stability selection, 8 were previously selected in relation to severity when all patients were used. None of the molecules selected in relation to COVID-19 status were selected. Compared with the analysis using all patients we end up with only two repeat pathways, which are the airway inflammation in asthma pathway and thyroid hormone metabolism II pathway. The new pathways selected are pathogenesis of multiple sclerosis, agranulocyte adhesion and diapedesis, as well as the complement system. These pathways are all related to the inflammatory response, once again highlighting the unique immune response to COVID-19. We have only one pathway with more than one selected molecule, so we look further into the agranulocyte adhesion and diapedesis pathway through a Pearson’s correlation coefficient between the PCs and clinical covariates (Fig 9). The agranulocyte adhesion and diapedesis pathway was found to have strong correlations with white blood cell count, as to be expected considering this pathway is directly involved in white blood cell production [29]. There is also a strong correlation between the PCs and different comorbidity scoring methods (SOFA, APACHE II, Charlson comorbidity index) methods. We also assess Agranulocyte Adhesion and Diapedesis pathways relationship with COVID-19 severity by fitting a linear regression model of HFD-45 on the first two PCs, adjusted for some clinical covariates, using only patients with COVID-19. The p-values of the different predictors are presented in Table 15. This pathway seems to play a significant role in COVID-19 severity outcome, along with age which is to be expected. From the regression results, the Charlson comorbidity index score is not statistically significant; this is likely due to the strong correlation with the first principal component. Further, the top affected biological functions reveal thirteen molecules that are related to dermatological diseases and conditions. This finding agrees with studies that found that severe cases of COVID -19 result in many patients experiencing dermatological symptoms [30].

**Table 14:** Enrichment analysis of genes associated with COVID-19 severity (patients with COVID-19).

Top Diseases and Biological Functions		
	P-value range	Selected Molecules
Dermatological Diseases and Conditions	2.71E-02–2.16E-04	*APBA1*, *C8B*, *CXCL9*, *CXXC4*, *ITGB4*, *LRGUK*, *MADCAM1*, *NFIB*, *PI3*, *RIPK4*, *SPATA20*, *SYNDIG1L*, *UGT2B11*
Organismal Injury and Abnormalities	4.99E-02–2.16E-04	*APBA1*, *C8B*, *CXCL9*, *CXXC4*, *GOLGA8T*, *ITGB4*, *LRGUK*, *MADCAM1*, *NFIB*, *PI3*, *RIPK4*, *RNASE2*, *SPATA20*, *SYNDIG1L*, *UGT2B11*
Renal and Urological Disease	2.43E-02–2.54E-04	*CXCL9*, *ITGB4*
Connective Tissue Disorders	4.71E-02–7.22E-04	*NFIB*, *ITGB4*, *CXCL9*, *RNASE2*
Developmental Disorder	4.94E-02–7.22E-04	*NFIB*, *RIPK4*, *C8B*, *ITGB4*
Cell-To-Cell Signalling and Interaction	4.11E-02–3.93E-05	*ITGB4*, *MADCAM1*, *PI3*, *CXCL9*, *RNASE2*, *APBA1*
Cell Cycle	2.29E-02–7.22E-04	*ITGB4*, *IP3*
Cell Morphology	4.87E-02–7.22E-04	*ITGB4*, *CXCL9*
Cellular Assembly and Organization	4.87E-02–7.22E-04	*ITGB4*, *MADCAM1*, *APBA1*, *CXCL9*
Cell Death and Survival	3.76E-02–1.44E-03	*ITGB4*, *CXCL9*, *RNASE2*, *PI3*
**Top Canonical Pathways**		
**Pathway**	**P-value**	**Selected Molecules**
Pathogenesis of Multiple Sclerosis	6.48E-03	*CXCL9*
Agranulocyte Adhesion and Diapedesis	6.97E-03	*CXCL9*, *MADCAM1*
Thyroid Hormone Metabolism II	2.22E-02	*UGT2B11*
Airway Inflammation in Asthma	2.22E-02	*RNASE2*
Complement System	2.57E-02	*C8B*

This table contains the enrichment analysis results for genes associated with disease severity when using patients with COVID-19 only. For the gene set enrichment analysis the IPA output contains the top five canonical pathways and top ten biological functions and disease associations.

**Table 15:** Regression models for genes associated with COVID-19 severity (all patients).

Pathway		(Intercept)	PC1	PC2	Age	Gender (Male)	Charlson Score
**Agranulocyte Adhesion and Diapedesis**	**Coef (SE)**	40.446 (5.841)	-6.515 (1.407)	-5.438 (1.499)	-0.288 (0.114)	-2.010 (2.830)	-0.108 (0.792)
	p-values *LRT	-	**<2e-16**	**0.001**	**<2e-16**	0.486	0.891
	**95% Conf.int**	(28.998, 51.895)	(-9.271, -3.758)	(-8.375, -2.500)	(-0.512, -0.064)	(-7.58, 3.537)	(-1.661, 1.444)
	**%Var Explained**	-	**57.02%**	**42.98%**	-	-	-

Summary of multivariate linear regression models with COVID-19 severity as the outcome using COVID-19 patients only, and the principal components used to summarize the enriched pathways associated with disease severity status as the predictors. The models are also adjusted for the clinical covariates age, sex and Charlson comorbidity score. P-values for significance are determined via the likelihood ratio test (LRT).

**Fig 10:**
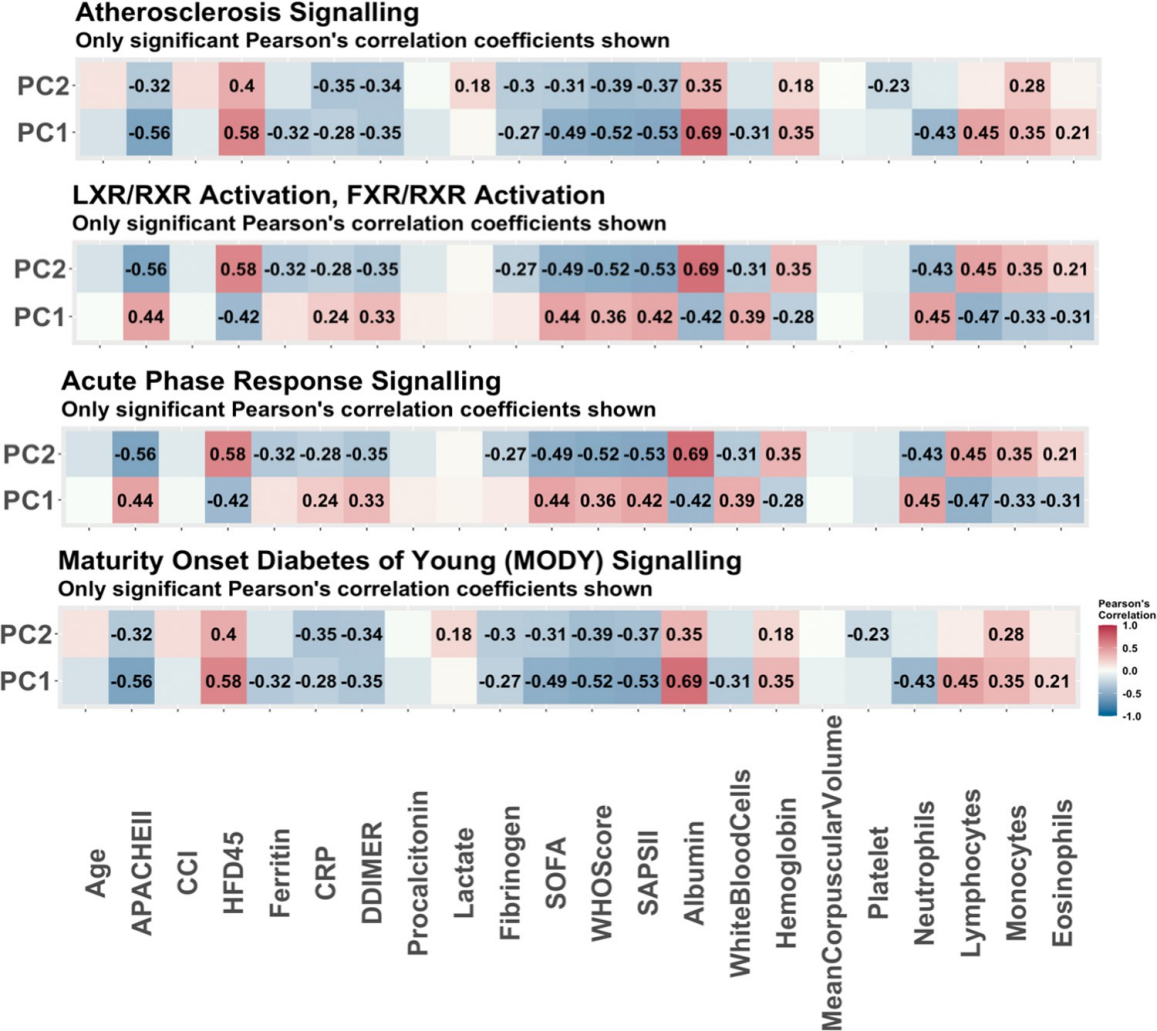
The Pearson correlations of principal components of pathways associated with disease severity (COVID-19 patients). The Pearson correlations of the principal components used to summarize the enriched pathways with COVID-19 severity as predicted by IPA. These are the pathways predicted to be enriched based on the 62 proteins determined to be significant in COVID-19 severity via stability selection which were able to be mapped to known pathways. Only the correlations which were significant (p-value<0.05) are reported. These pathways have many strong correlations with clinical covariates.

**Fig 11:**
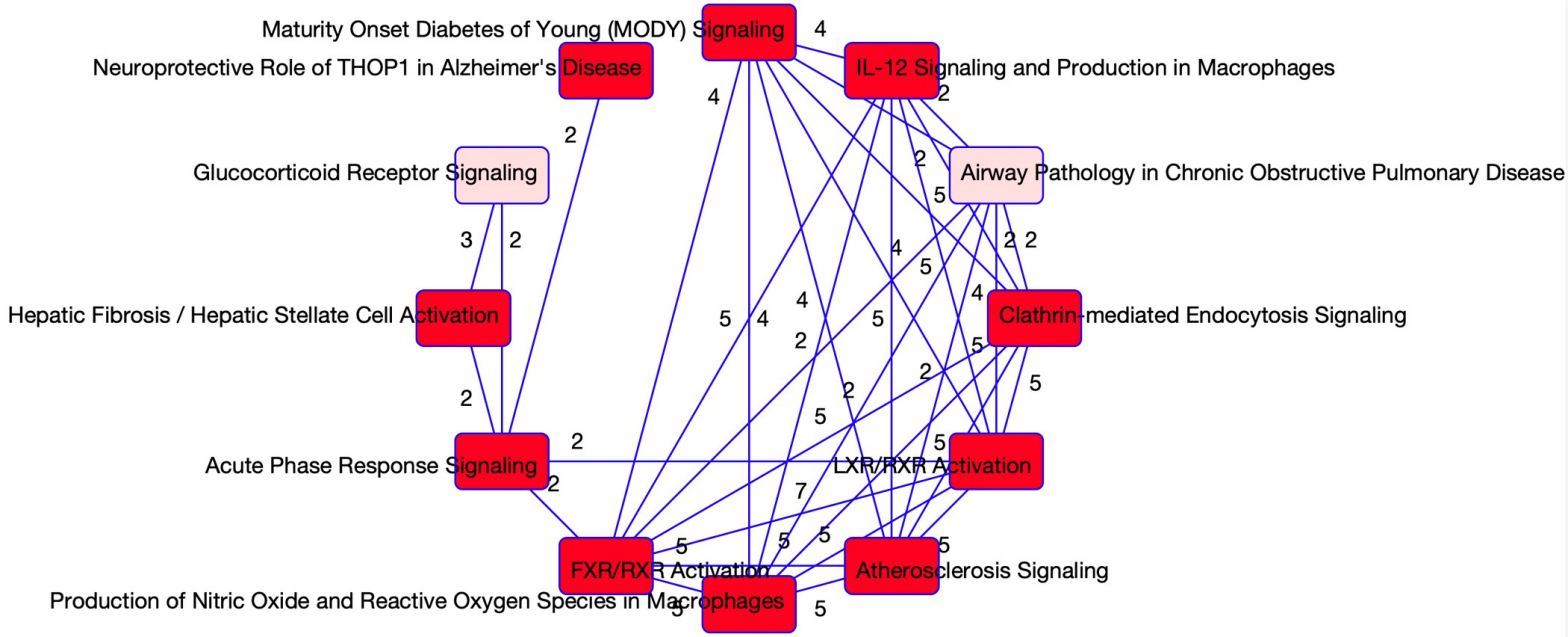
Overlapping networks associated with COVID-19. Visual of the overlapping networks enriched in COVID-19 as determined from the proteomics data. The nodes represent the networks and the edges represent the overlapping genes between the networks. The edge labels provide us with the number of overlapping molecules between the networks.

There were 64 proteins selected via the stability selection methodology to be associated with COVID-19 severity, however 2 of the molecules were unable to be mapped to any pathways. Of the 64 proteins, 2 were selected previously in relation to COVID-19 status. The output from the IPA is summarized in Table 16 and the enriched pathways were further analyzed via PCA, and regression models. The FXR/RXR Activation and LXR/RXR Activation pathways selected the same molecules so we fit one model. Because these molecules were selected specifically looking at the disease severity of patients with COVID-19, the regression models are fit using only the 99 COVID-19 patients. The results of correlations with clinical covariates are available in Fig 10 while the results of the regression models are available in Table 17. We find that the Charlson score has significance, however we observe that the pathway’s PCs still tend to be more significant with the exception of the FXR/RXR and LXR/RXR activation pathways. Four of the enriched pathways were also found to be enriched when the full sample was used. The maturity onset diabetes of young (MODY) signaling pathway was enriched in the analysis of data from COVID-19 patients only. MODY develops slowly and impairs insulin secretion so that the body cannot adequately control blood glucose levels [31]. This relationship is interesting as it aligns with current studies that individuals with diabetes are experiencing more severe symptoms of COVID-19. A visual of the overlapping networks is found in Fig 11.

**Table 16:** Enrichment analysis of proteins associated with COVID-19 severity (patients with COVID-19).

Top Diseases and Biological Functions		
	P-value range	Selected Molecules
Inflammatory Response	1.69E-02–2.27E-11	*ACSL6*, *AGT*, *APOA2*, *APOM*, *B4GALT1*, *CD163*, *CHI3L1*, *DEFA1 HBA1/HBA2*, *HBB*, *HLA-C*, *HSPD1*, *ICAM1*, *IGKV1D-8*, *IGHV1OR15-1*, *IGKV1-12*, *IGLC7*, *ITIH3*, *JCHAIN*, *LTA4H*, *MBL2*, *MCAM*, *MRC1*, *PIGR*, *PPBP*, *PVR*, *PYGL*, *SERPINA3*, *SERPINE1*, *SPARC*, *SPP2*, *THBS4*, *TNC*, *TNXB*, *TUBB*
Infectious Diseases	1.69E-02–2.05E-09	*APOA2*, *APOD*, *APOM*, *B4GALT1*, *DEFA1*, *HBA1/HBA2*, *HBB*, *HLA-C*, *HSPD1*, *ICAM1*, *ITIH3*, *LTA4H*, *MBL2*, *MCAM*, *MRC1*, *PCYOX1*, *PPBP*, *PVR*, *PYGL*, *SERPINA3*, *SERPINE1*, *SFTPB*, *SPARC*, *TKT*, *TUBB*
Metabolic Disease	1.30E-02–4.96E-07	*AGT*, *APOA2*, *APOC4-APC2*, *APOD*, *APOM*, *B4GALT1*, *CHI3L1*, *CHL1*, *COL6A1*, *FBLN5*, *GNPTG*, *HBA1/HBA2*, *HBB*, *HLA-C*, *HSPD1*, *ICAM1*, *IGFBP7*, *MBL2*, *MRC1*, *PYGL*, *SFTPB*, *SERPINA3*, *SERPINE1*, *SPARC*, *TKT*, *TNC*, *TNXB*, *TUBB*
Neurological Disease	1.69E-02–4.96E-07	*ACSCL6*, *AGT*, *ANK1*, *APOA2*, *APOD*, *APOF*, *CHI3L1*, *CHL1*, *DBH*, *FBLN5*, *HBA1/HBA2*, *HBB*, *HSPD1*, *ICAM1*, *MCAM*, *MRC1*, *NCAM1*, *OIT3*, *OLFM1*, *PYGL*, *PZP*, *SERPINA3*, *SERPINE1*, *SPARC*, *SPARCL1*, *THBS4*, *TNC*, *TNXB*, *TUBB*, *WARS1*, *ZSWIM9*
Organismal Injury and Abnormalities	1.97E-02–4.96E-07	*ACSL6*, *AGT*, *ANK1*, *APOA2*, *APOC4-APOC2*, *APOD*, *APOM*, *APOF*, *B4GALT1*, *CD163*, *CD5L*, *CHI3L1*, *CHL1*, *COL6A1*, *DBH*, *DEFA1*, *FBLN5*, *GNPTG*, *HBA1/HBA2*, *HBB*, *HLA-C*, *HSPD1*, *ICAM1*, *IGFBP7*, *IGKV1D-8*, *ITIH3*, *JCHAIN*, *LTA4H*, *MBL2*, *MBL2*, *MCAM*, *MRC1*, *NCAM1*, *NID1*, *OLFM1*, *OIT3*, *PCYOX1*, *PIGR*, *PON3*, *PPBP*, *PROZ*, *PVR*, *PYGL*, *PZP*, *SERPINE1*, *SERPINA3*, *SFTPB*, *SPARC*, *SPARCL1*, *TKT*, *THBS4*, *TNC*, *TNXB*, *TUBB*, *WARS1*, *ZSWIM9*
Cellular Compromise	1.41E-02–2.27E-11	*B4GALT1*, *CHI3L1*, *DEFA1*, *HBB*, *HLA-C*, *ITIH3*, *LTA4H*, *MCAM*, *PIGR*, *PPBP*, *PVR*, *PYGL*, *SERPINA3*, *SERPINE1*, *SPARC*, *SPP2*, *TUBB*
Cellular Movement	1.69E-02–6.06E-11	*AGT*, *APOD*, *CHI3L1*, *CHL1*, *COL6A1*, *DEFA1*, *FBLN5*, *HSPD1*, *ICAM1*, *IGFBP7*, *IGHV1OR15-1*, *IGKV1-12*, *IGLC7*, *JCHAIN*, *MCAM*, *NCAM1*, *PIGR*, *PPBP*, *PVR*, *SERPINA3*, *SERPINE1*, *SPARC*, *SPARCL1*, *THBS4*, *TNC*, *WARS*
Cellular Function and Maintenance	1.01E-02–7.52E-10	*APOA2*, *ANK1*, *CD163*, *CD5L*, *HBA1/HBA2*, *HBB*, *ICAM1*, *IGHV1OR15-1*, *IGKV1-12*, *IGLC7*, *JCHAIN*, *MBL2*, *MCAM*, *MRC1*, *PIGR*, *SERPINE1*, *SPARC*, *THBS4*
Cell-To-Cell Signalling and Interaction	1.97E-02–1.30E-06	*AGT*, *B4GALT1*, *DBH*, *CD163*, *DEFA1*, *ICAM1*, *IGFBP7*, *MBL2*, *MCAM*, *MRC1*, *NCAM1*, *NID1*, *PIGR*, *PPBP*, *SERPINE1*, *SFTPB*, *SPARC*, *SPARCL1*, *THBS4*, *TNC*
Protein Synthesis	8.50E-03–2.58E-06	*AGT*, *APOA2*, *HSPD1*, *IGFBP7*, *LTA4H*, *PCYOX1*, *SFTPB*, *SPARCL1*, *SPP2*, *TNC*, *WARS1*
**Top Canonical Pathways**		
**Pathway**	**P-value**	**Selected Molecules**
LXR/RXR Activation	5.07E-08	*AGT*, *APOA2*, *APOD*, *APOF*, *APOM*, *PCYOX1*, *PON3*
FXR/RXR Activation	6.35E-08	*AGT*, *APOA2*, *APOD*, *APOF*, *APOM*, *PCYOX1*, *PON3*
Atherosclerosis Signalling	1.33E-06	*APOA2*, *APOD*, *APOF*, *APOM*, *ICAM1*, *PCYOX1*
Acute Phase Response Signalling	1.16E-05	*AGT*, *APOA2*, *ITIH3*, *MBL2*, *SERPINA3*, *SERPINE1*
Maturity Onset of Young Diabetes Signaling (MODY)	2.38E-05	*APOA2*, *APOD*, *APOF*, *APOM*

This table contains the enrichment analysis results for proteins associated with disease severity when using patients with COVID-19 only. For the protein set enrichment analysis the IPA output contains the top five canonical pathways and top ten biological functions and disease associations.

**Table 17:** Enrichment analysis of proteins associated with COVID-19 severity (patients with COVID-19).

Pathway		(Intercept)	PC1	PC2	Age	Gender (Male)	Charlson Score
**LXR/RXR Activation/FXR/RXR Activation**	**Coef (SE)**	36.911 (6.645)	-3.915 (2.181)	5.363 (2.418)	-0.135 (0.132)	-2.315 (3.225)	-1.561 (0.836)
	p-values *LRT	-	**0.003**	**0.010**	**0.002**	0.741	0.062
	**95% Conf.int**	(23.886, 49.936)	(-8.190, 0.361)	(0.623, 10.102)	(-0.393, 0.123)	(-8.636, 4.005)	(-3.199, 0.076)
	**%Var Explained**	-	**51.64%**	**16.63%**	-	-	-
**Atherosclerosis Signalling**	**Coef (SE)**	37.090 (5.250)	-4.756 (0.671)	-5.971 (1.460)	-0.116 (0.103)	-3.809 (2.569)	-1.208 (0.677)
	p-values *LRT	-	**<2e-16**	**<2e-16**	**0.002**	0.245	0.075
	**95% Conf.int**	(26.801, 47.379)	(-6.072, -3.441)	(-8.850, -3.092)	(-0.319, 0.086)	(-8.844, 1.226)	(-2.535, 0.119)
	**%Var Explained**	-	**58.49%**	**16.98%**	-	-	-
**Acute Phase Response Signalling**	**Coef (SE)**	38.192 (6.196)	2.337 (0.849)	3.028 (0.903)	-0.106 (0.122)	-1.899 (3.041)	-2.446 (0.818)
	p-values *LRT	**-**	**0.029**	**0.001**	**<2e-16**	0.921	**0.003**
	**95% Conf.int**	(26.048, 50.336)	(0.674, 4.000)	(1.259, 4.797)	(-0.346, 0.134)	(-7.860, 4.062)	(-4.049, -0.843)
	**%Var Explained**	-	**39.37%**	**34.47%**	-	-	-
**Maturity Onset Diabetes of Young (MODY) Signalling**	**Coef (SE)**	37.491 (6.121)	-8.948 (1.278)	-1.317 (2.195)	-0.107 (0.113)	-4.442 (2.771)	-1.292 (0.708)
	p-values *LRT	-	**<2e-16**	**0.009**	**0.013**	0.210	0.068
	**95% Conf.int**	(25.494, 49.487)	(-11.453, -6.443)	(-5.619, 2.985)	(-0.330, 0.115)	(-9.872, 0.989)	(-2.679, 0.095)
	**%Var Explained**	-	**59.65%**	**18.97%**	-	-	-

Summary of multivariate linear regression models with COVID-19 severity as the outcome using patients with COVID-19 only, and the principal components used to summarize the enriched pathways associated with disease severity status as the predictors. The models are also adjusted for the clinical covariates age, sex and Charlson comorbidity score. P-values for significance are determined via the likelihood ratio test (LRT).

The same metabolites were found in stability selection when the COVID-19 subset was used as when the full sample was used, and the ranking of the metabolites was so similar that the IPA results are the same.

Of the 31 stability selected lipids which were significant in COVID-19 severity, only 9 were able to be mapped to pathways. The top 5 significant pathways are presented in Table 18. None of the pathways contain more than one molecule so PCA was not performed. We note a recurring theme here again of an improperly regulated cell cycle as ferroptosis is associated with severity which is a form of programmed cell death.

**Table 18:** Enrichment analysis of lipids associated with COVID-19 severity (COVID-19 patients).

Enriched Pathways				
Pathway	p-value	Benjamini correction	Bonferroni correction	Selected Molecules
**Sphingolipid metabolism**	6.94137E-06	0.000104121	0.000104121	*HexCer36*:*2;O2*, *HexCer40*:*2;O2*, *HexCer44*:*0;O2*, *SPBP18*:*1;O2 *
**Fat digestion and absorption**	0.018723372	0.084269663	0.280850581	*CE*.*18*.*0*
**Cholesterol metabolism**	0.018723372	0.084269663	0.280850581	*CE*.*18*.*0*
**Sphingolipid signaling pathway**	0.023682416	0.084269663	0.355236233	*SPBP18*:*1;O2*
**Basal cell carcinoma**	0.028089888	0.084269663	0.421348315	*CE*.*18*.*0*

This table contains the enrichment analysis results for lipids associated with disease severity when using patients with COVID-19 only. For the lipidomics set enrichment analysis from LIPEA the top 10 enriched pathways are summarized.

### 3.4 Unsupervised integrative analysis

**Fig 12:**
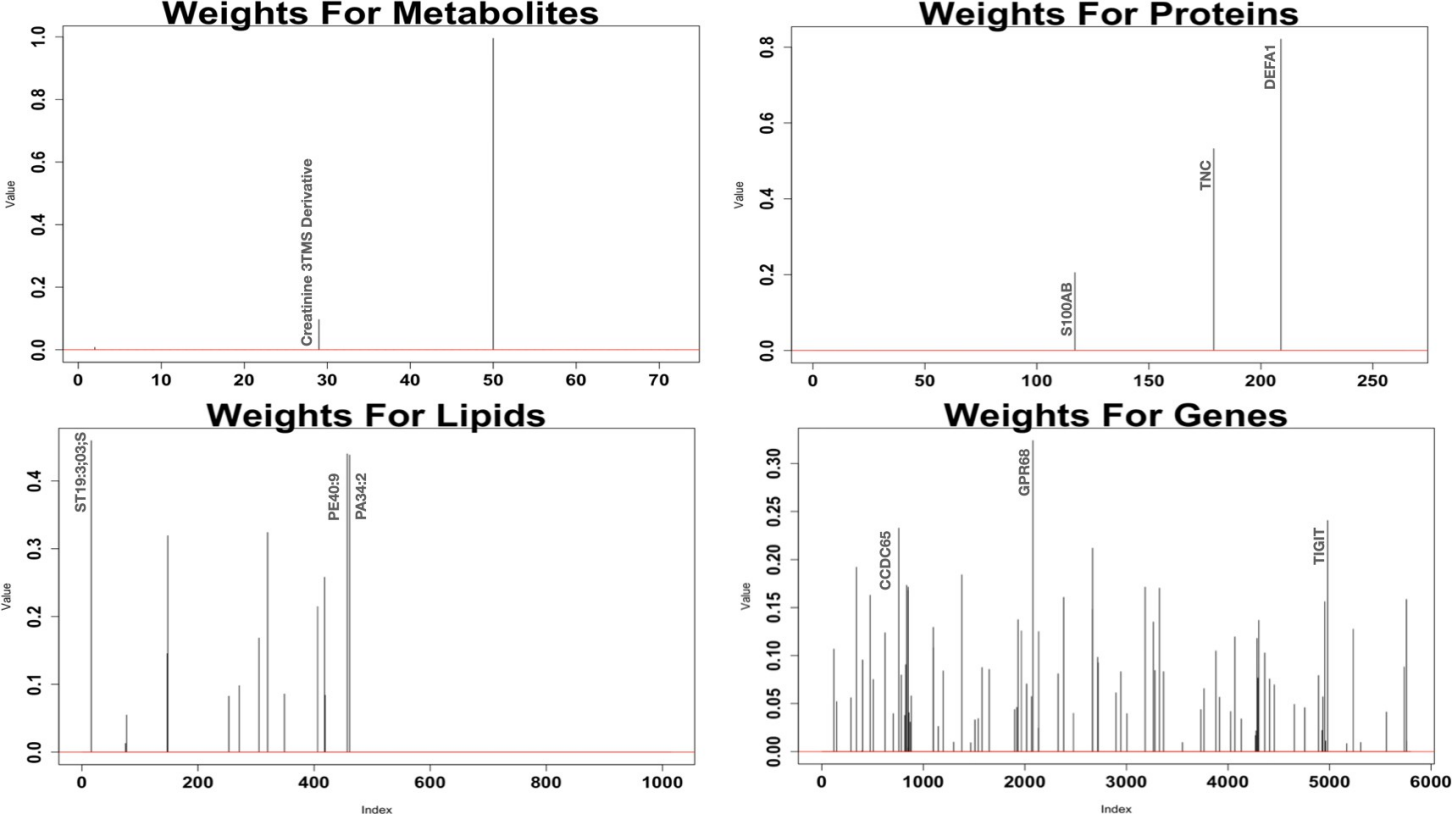
Absolute coefficients for first component of smCCA. These plots contain the absolute values of the weights for each datasets’ first component in smCCA.

**Fig 13:**
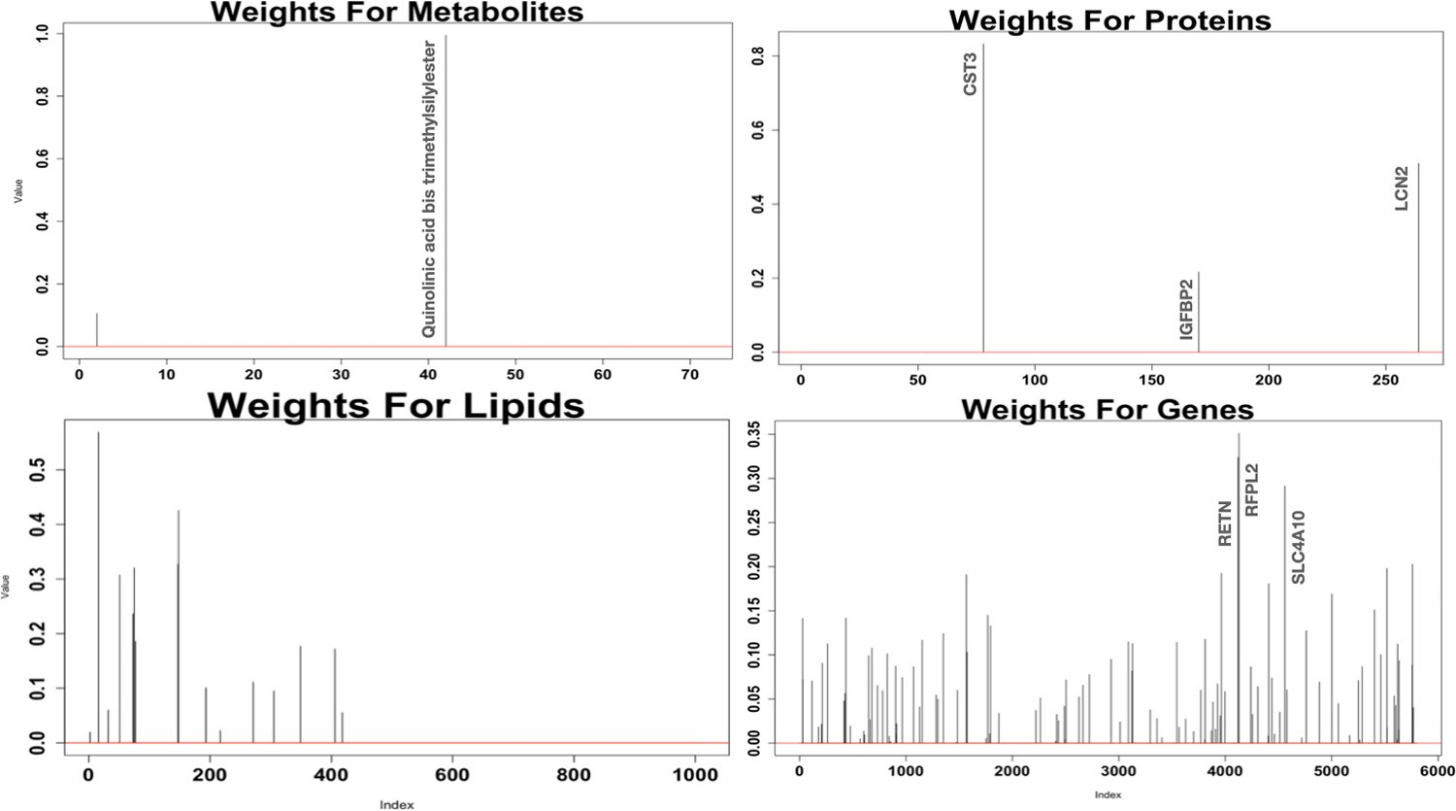
Absolute coefficients for second component of smCCA. These plots contain the absolute values of the weights for each datasets’ second component in smCCA.

**Fig 14:**
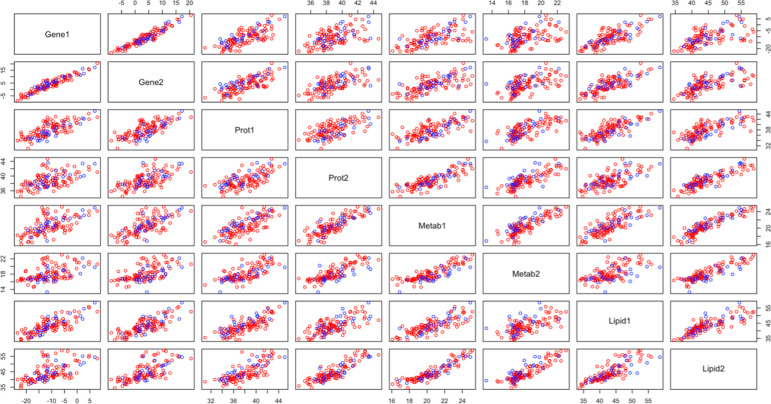
smCCA scores. Plot of the pairwise scores from smCCA. Note the red points are patients with COVID-19 and the blue are patients without.

From the unsupervised integrative analysis we look at two components which are able to capture the correlation structure, for each dataset plots of the absolute values of the coefficients for the first component are available in Fig 12, and from the second components in Fig 13. It is observed that the datasets with the most molecules selected are the lipidomics and genomics data. The molecules selected from this analysis are compared with the molecules selected via stability selection. The lipid PA34:2, which was selected in component 2, is the only lipid which had previously been selected in stability selection. The only annotated metabolite selected for component 2, Quinolinic acid 2TMS derivative, was selected for all three stability selections performed on metabolites. Violin plots of these particular molecules are available in S4 and S5 Figs in S1 File. The protein with gene name TNC, which was selected in component 1, was previously also selected in stability selection and a violin plot for this particular molecule is available in S6 Fig in S1 File. There was no overlap with the stability selected genes and the selected genes in smCCA. Plots of the 3 genes with the largest coefficients for component 1 and component 2 are provided in S7 and S8 Figs in S1 File respectively. IPA was performed individually on the 88 genes selected in component 1, and the 111 molecules selected in component 2 from the analysis. The results of these analyses are available in Tables 19 and 20 respectively. The top canonical pathways from both components were not pathways previously determined to be enriched using the stability selected molecules, however we observe that many of the top biological functions and diseases were previously also determined to be enriched. It is of interest to determine the pairwise correlations of the datasets. The pairwise correlations of the scores from the smCCA are available in Table 21. From the table, the strongest associations are between the metabolomics and lipidomics, as well as the metabolomics and proteomics data. All the correlations are quite strong, with only one of the correlations being less than 0.5. To visually assess whether this unsupervised method is able to separate patients by COVID-19 status, we provide scatter plots of the component scores for each dataset in Fig 14. From these plots it appears that the pairwise component scores are not able to accurately separate the patients according to disease status. To assess statistically whether using all components can differentiate patients by disease status, we perform a simple logistic regression. The results of the regression are available in Table 22. From the regression, the metabolomics and proteomics score are most significant in COVID-19 status. Also, the components are 87.8% accurate at identifying true positive COVID-19 cases.

**Table 19:** Enrichment analysis of genes in smCCA component 1.

Top Diseases and Biological Functions		
	P-value range	Number of Molecules
Gastrointestinal Disease	2.06E-02–7.14E-11	80
Organismal Injury and Abnormalities	2.06E-02–7.14E-11	87
Cancer	2.06E-02–1.18E-09	87
Hematological Disease	2.06E-02–1.18E -09	42
Immunological disease	2.06E-02–1.18E -09	52
Cellular Compromise	2.06E-02–6.90E-08	14
Cellular Movement	2.06E-02–1.36E-07	28
Cellular Function and Maintenance	1.01E-02–1.36E-07	8
Cell-To-Cell Signalling and Interaction	2.06E-02–3.43E-07	31
Protein Synthesis	2.06E-02–4.29E-07	22
**Top Canonical Pathways**		
**Pathway**	**P-value**	**Selected Molecules**
TH1 Pathway	1.37E-09	*CD247*, *CD3D*, *CD3E*, *CD3G*, *CD8A*, *GATA3*, *NFATC2*, *PRKCQ*, *TBX21*
TH1 and TH2 Activation Pathway	1.86E-09	*CD247*, *CD3D*, *CD3E*, *CD3G*, *CD8A*, *GATA3*, *NFATC2*, *PRKCQ*, *TBX21*, *TGFBR3*
TH2 Pathway	4.28E-09	*CD247*, *CD3D*, *CD3E*, *CD3G*, *GATA3*, *NFATC2*, *PRKCQ*, *TBX21*, *TGFBR3*
Natural Killer Cell Signalling	11.09E-07	*CD247*, *FYN*, *KLRC4-KLRK1/KLRK1*, *LCK*, *NCR3*, *NFATC2*, *PLCG1*, *PRKCQ*, *SH2D1A*
T-Cell Receptor Signalling	4.55E-07	*CD247*, *CD3D*, *CD3E*, *CD3G*, *CD8A*, *FYN*, *LCK*, *NFATC2*, *PLCG1*, *PRKCQ*, *SKAP1*

This table contains the enrichment analysis results for genes active in component 1 from smCCA For the gene set enrichment analysis the IPA output contains the top five canonical pathways and top ten biological functions and disease associations.

**Table 20:** Enrichment analysis of genes in smCCA component 2.

Top Diseases and Biological Functions		
	P-value range	Number of Molecules
Cancer	3.07E-02–6.78E-06	109
Organismal Injury and Abnormalities	3.07E-02–6.78E-06	109
Gastrointestinal Disease	2.67E-02–9.89E-06	101
Inflammatory Response	2.68E-02–5.90E-05	16
Dermatological Disease and Conditions	3.07E-02–6.48E-04	84
Cell Death and Survival	3.07E-02–4.90E-05	21
Cell-To-Cell Signalling and Interaction	2.68E-02–5.90E-05	26
Cellular Growth and Proliferation	2.57E-02–6.81E-05	16
Cellular Development	2.38E-02–5.20E-04	18
Lipid metabolism	3.07E-02–2.33E-03	9
**Top Canonical Pathways**		
**Pathway**	**P-value**	**Selected Molecules**
Cellular Effects of Sildenafil	1.37E-09	*CACNA2D2*, *CAMK4*, *ITPR3*, *MPRIP*, *PRKACB*, *SLC4A10*
Netrin Signaling	1.86E-09	*ABLIM1*, *CACNA2D2*, *ITPR3*, *PRKACB*
Chemokine Signaling	4.28E-09	*CAMK4*, *CCL4*, *MPRIP*, *RRAS2*
Role of NFAT in Cardiac Hypertrophy	11.09E-07	*AKAP5*, *CACNA2D2*, *CAMK4*, *ITPR3*, *PRKACB*, *RRAS2*
Role of NFAT in Cardiac Hypertrophy	4.55E-07	*BCL2*, *CACNA2D2*, *CAMK4*, *ITPR3*, *PRKACB*

This table contains the enrichment analysis results for genes active in component 2 from smCCA For the gene set enrichment analysis the IPA output contains the top five canonical pathways and top ten biological functions and disease associations.

**Table 21:** Pairwise correlations of smCCA scores.

	Genes (1)	Genes (2)	Prot (1)	Prot (2)	Metab (1)	Metab (2)	Lipid (1)	Lipid (2)
**Genes (1)**	1	0.95	0.75	0.54	0.62	0.49	0.79	0.6
**Genes (2)**	0.95	1	0.75	0.64	0.7	0.58	0.78	0.68
**Prot (1)**	0.75	0.75	1	0.66	0.75	0.72	0.78	0.76
**Prot (2)**	0.54	0.64	0.66	1	0.83	0.8	0.69	0.88
**Metab (1)**	0.62	0.7	0.75	0.83	1	0.84	0.8	0.92
**Metab (2)**	0.49	0.58	0.72	0.8	0.84	1	0.63	0.88
**Lipid(1)**	0.79	0.78	0.78	0.69	0.8	0.63	1	0.82
**Lipid(2)**	0.6	0.68	0.76	0.88	0.92	0.88	0.82	1

Pairwise correlations of scores from smCCA.

**Table 22:** Logistic regression using smCCA components.

	Coef (SE)	p-values *LRT		True COVID-19	True Non-Covid-19	
**(Intercept)**	2.773 (10.626)	-	**Pred. COVID-19**	96	12	
**Age**	0.000 (0.019)	0.518	**Pred. Non -COVID-19**	3	12	
**Sex(Male)**	-1.739 (0.672)	0.279				
**Genes (1)**	-0.024 (0.173)	0.212				
**Genes (2)**	0.235 (0.201)	0.179			
**Prot (1)**	-0.404 (0.219)	**0.004**			
**Prot (2)**	0.424 (0.337)	0.076			
**Metab (1)**	1.035 (0.447)	0.246			
**Metab (2)**	-1.710 (0.466)	**<2e-16**			
**Lipid(1)**	-0.066 (0.147)	0.911			
**Lipid(2)**	0.140 (0.192)	0.466			

Results from logistic regression using components from smCCA. Significant p-values at a level of 0.05 are bolded.

## 4. Discussion

From these independent omics analyses we notice some unique patterns and signatures of COVID-19 emerge. Specifically, when looking at associations with disease status we realize a dysregulated cell cycle reflected in the RNAseq enrichment analysis. This dysregulated system is also apparent in the proteomics and metabolomics analyses where we observe several of the selected molecules to be related to cell function and survival. From these independent analyses it is also apparent there is an association with neurological conditions and COVID-19 as reflected in the 22 molecules determined to be significant from the proteomics, lipidomics, and RNAseq data. In addition, the proteomics, metabolomics, and lipidomics datasets indicate that regulation and activation of metabolic processes, especially of cholesterol and vitamins, are significantly associated with COVID-19 status and severity. This gives us a broad insight into the signature of the disease. When looking at disease severity we discern a common theme across all the datasets of an association with comorbidities such as diabetes as reflected in the proteomics data, cancer which is reflected in the RNAseq data where 23 cancer associated molecules are chosen, as well as the lipidomics data. Associated with disease status and disease severity we also have molecules involved with dermatological conditions. This is reflected in both the RNAseq enrichment analysis and the metabolomics enrichment analysis. This association corroborates other studies which have demonstrated long term dermatological symptoms associated with severe COVID-19 cases. It is apparent from this analysis that COVID-19 is a disease which disrupts many biological systems, and the unique relationships to diseases such as dermatological and neurological conditions could mean serious implications for individuals who have been infected with the disease. These associations should be further analyzed to better understand the effects and develop treatments. The unsupervised integrative method used is able to capture the correlation structure of the datasets and provide a set of important molecules. The analysis of these molecules via IPA again displayed molecules associated with cancer and enriched in COVID-19. For future research we will be conducting a supervised integrative analysis of all the data sets and clinical data to get a broader perspective on the disease and enriched pathways. A supervised integrative method will allow us to assess associations across the datasets while considering clinical outcomes. These associations have been hinted at in the independent analysis with recurring molecular themes and confirmed through smCCA.

## Supporting information

S1 File Contains all the supporting tables and figures. (DOCX)
