# Supplementary material for: Multi-omic analysis reveals enriched pathways associated with COVID-19 and COVID-19 severity
Source: PLoS One. 2022 Apr 25;17(4):e0267047. doi: 10.1371/journal.pone.0267047 (PMC9038205; doi:10.1371/journal.pone.0267047)
Supplement: S1 File — (DOCX) [file pone.0267047.s001.docx]

# Supplementary Material

**S1 Table**

**Clinical covariates**

A summary of the clinical variables initially collected in the Overmeyer et al., 2020 paper. None of the variables with missing data are considered in our regression analyses.

| **Variable** | **Description** | **# Missing** |
| --- | --- | --- |
| **Disease Status** | An indicator variable of the patient’s COVID-19 status. | 0 |
| **Sex** | Sex of the patient. 12/24 of the non-COVID-19 patients are male, and 61/99 of the COVID-19 are male | 0 |
| **ICU Status** | Indicator variable of the patient’s ICU status. | 0 |
| **Ventilator Free Days** | Out of the 45 days enrolled in the study, the number of ventilator-free days is recorded |  |
| **APACHE II Score** | Acute Physiologic Assessment and Chronic Health Evaluation II generates a point score ranging from 0 (best health) to 71 (worst health) based on physiologic variables, age, and health conditions. | 51 |
| **Charlson Score** | A score to assess the comorbidities of a patient based on number and severity of comorbid conditions with higher scores indicating more comorbidities. | 0 |
| **Mechanical**  **Ventilation Status** | Indicator variable of the patient’s mechanical ventilation status. | 0 |
| **HFD45** | Out of the 45 days enrolled in the study, the number of hospital-free days is recorded with 0 indicating the patient was still admitted after 45 days or they had died. | 0 |
| **SOFA Score** | The Sequential Organ Failure Assessment **Score** predicts ICU mortality based on lab results and clinical data with a higher score indicating a higher mortality rate. | 51 |
| **WHO Score** | WHO ordinal score from 0–8 (8 denotes death) captures COVID-19 specific severity metrics and mortality (World Health Organization, 2020). The WHO ordinal score is taken at 28 days | 0 |
| **Days Before Enrolment** | The amount of days the patients were admitted to the hospital before enrolment in the study. | 0 |

**S2 Table**

**Stability selected molecules**

This table contains the lists of molecules selected via stability selection.

| **Selection Criteria** | **List of Molecules** |
| --- | --- |
| **Genes Significant for COVID-19 Status** | *CCNB1, CDC25C, CDC45, CDC6, DEPDC1B, DIAPH3, FAM3D, FHL2, MCM10, MND1, OBSL1, PBK, RNA28SN4, RNA5S1, TYMS* |
| **Genes Significant for Disease Severity (All Patients)** | *C17orf97, CNR1, CXXC4, DNAAF1, EPAS1, GOLGA8T, HEPHL1, LOC441155, LRGUK, MEIS3, MFAP4, MYO5B, NECAB2, NFIB, OR52N4, PRSS50, RBP5, RIPK4, RNASE2, SEZ6L, SMIM33, SPATA20, TMEM52B, UGT2B11, ZNF221* |
| **Genes Significant for Disease Severity (Patients with COVID-19 only)** | *APBA1, C2orf66, C8B, CXCL9, CXXC4, GOLGA8T, ITGB4, TP53TG3F, LRGUK, MADCAM1, NFIB, PI3, RIPK4, RNASE2, SPATA20, SYNDIG1L, UGT2B11* |
| **Proteins Significant for COVID-19 Status (Gene Symbol)** | *APMAP, C4A, CKM, CRTAC1, CSF1R, H3C15, HLA-B, HRG, HSPD1, IGHV1-2, IGHV3-20, IGHV3-21, IGKV2-30, P01715, LCP1, LGALS3BP, LUM, PLTP, SFTPB, TTR* |
| **Proteins Significant for Disease Severity (All Patients) (Gene Symbol)** | *IGHV1OR15, CHL1, IGHV1-3, IGHV3-38, IGHV2-70D, IGKV1D-8, CTSD, TNXB, IGLC7, APOD, ACSL6, SFTPB, THBS4, SERPINA3, FBLN5, HP, FCGR3A, COL18A1, APOC4-APOC2, HLA-C, CD5L, APOM, VNN1, AGT, JCHAIN, IGLV1-44, IGHV2-70, PPBP, HRG, HLA-A, DBH, LTA4H, C4A, IGHV1-8, IGHV4382, CETP, LCP1, NID1, ANPEP, B4GALT1, ANK1, PZP, PROZ, MRC1, WARS1, TNC, PSMA5, HLA-A, CHI3L1, CCT6A, MASP1, HBB, HBA1, CFHR1, ITIH3, SPP2, APOF, SPARCL1, PON3, IGFBP7, TIMP1, OAF, ZSWIM9, CNDP1, PCYOX1, GNPTG, ANGPTL3, H2BC15, APOA2* |
| **Proteins Significant for Disease Severity (Patients with COVID-19 only) (Gene Symbol)** | *CHL1, COL6A1, PVR, IGHV1-, IGHV3-38, IGHV2-70D, IGKV1-12, IGKV1D-, TNXB, IGLC7, APOD, ACSL6, SFTPB, THBS4, CD163, SERPINA3, FBLN5, APOC4-APOC2, HLA-, CD5L, APOM, AGT, JCHAIN, PIGR, PPBP, SERPINE1, ICAM1, PYGL, DBH, SPARC, LTA4H, IGHV4-38-2, HSPD1, MBL2, NCAM1, NID1, B4GALT1, ANK1, PZP, PROZ, MRC1, WARS1, TNC, TKT, CHI3L1, MCAM, DEFA3, HBB,HBA1, ITIH3, SPP2, APOF, SPARCL1, PON3, IGFBP7, TUBB, ZSWIM9, OIT3, OLFM1, PCYOX1, GNPTG, APOA2* |
| **Metabolites Significant for COVID-19 Status** | *Methylphenol, 3-Hydroxybutyric acid 2TMS derivative, 2-Aminobutyric acid,*  *3-Hydroxyisovaleric acid 2TMS derivative, 2-Ketoisocaproic acid TMS derivative, Glycerol 3TMS derivative, L-Isoleucine 2TMS derivative, Glycine 3TMS derivative, Sucrose, Salicylic acid, Phenylalanine 2TMS derivative, Sucrose, Quinolinic acid 2TMS derivative, Sugar acid, Palmitic Acid TMS derivative, myo-Inositol, Naproxen TMS derivative, L-Kynurenine, L-Tryptophan 3TMS derivative, Stearic acid TMS derivative, Pseudo uridine penta-tms* |
| **Metabolites Significant for Disease Severity (All Patients)** | *2-Aminobutyric acid, 2-Ketoisocaproic acid TMS derivative, 3-Hydroxybutyric acid 2TMS derivative, 3-Hydroxyisovaleric acid 2TMS derivative, Glycerol 3TMS derivative, Glycine 3TMS derivative, L-Isoleucine 2TMS derivative, L-Kynurenine, L-Tryptophan 3TMS derivative, Methylphenol, myo-Inositol, Naproxen TMS derivative, Palmitic Acid TMS derivative, Phenylalanine 2TMS derivative, Pseudo uridine penta-tms, Quinolinic acid 2TMS derivative, Salicylic acid, Stearic acid TMS derivative, Sucrose, 2-Hydroxybutyric acid 2TMS derivative, Phosphoric acid polymer with 2-aminoethanol, Butanoic acid, 3,4-bis[(trimethylsilyl)oxy]-, trimethylsilyl ester, L-Aspartic acid, N-(trimethylsilyl)-, bis(trimethylsilyl) ester, (1-Methyl-3-(trimethylsilyl)-4-[(trimethylsilyl)oxy]-1,3-dihydro-2H-imidazol-2-ylidene)(trimethylsilyl)amine, Trimethylsilyl 2,3,4-tris[(trimethylsilyl)oxy]butanoate, Acetic acid, [o-(trimethylsiloxy)phenyl]-, trimethylsilyl ester, 1H-Indole, 1-(trimethylsilyl)-5-[(trimethylsilyl)oxy]-, 9H-Purine, 9-(trimethylsilyl)-6-[(trimethylsilyl)oxy]-, Sugar acid, Uric acid* |
| **Metabolites Significant for Disease Severity (Patients with COVID-19 only)** | *2-Hydroxybutyric acid 2TMS derivative, Methylphenol, 3-Hydroxybutyric acid 2TMS derivative, 2-Aminobutyric acid, 3-Hydroxyisovaleric acid 2TMS derivative, 2-Ketoisocaproic acid TMS derivative, Phosphoric acid, polymer with 2-aminoethanol, Glycerol 3TMS derivative, L-Isoleucine 2TMS derivative Glycine, 3TMS derivative, Butanoic acid, 3,4-bis[(trimethylsilyl)oxy]-, trimethylsilyl ester Sucrose, L-Aspartic acid, N-(trimethylsilyl)-, bis(trimethylsilyl) ester Salicylic acid, (1-Methyl-3-(trimethylsilyl)-4-[(trimethylsilyl)oxy]-1,3-dihydro-2H-imidazol-2-ylidene)(trimethylsilyl)amine, Trimethylsilyl 2,3,4-tris[(trimethylsilyl)oxy]butanoate, Phenylalanine 2TMS derivative, Acetic acid, [o-(trimethylsiloxy)phenyl]-, trimethylsilyl ester, 1H-Indole, 1-(trimethylsilyl)-5-[(trimethylsilyl)oxy]-, Quinolinic acid 2TMS derivative, 9H-Purine, 9-(trimethylsilyl)-6-[(trimethylsilyl)oxy]-, Sugar acid, Palmitic Acid TMS derivative, myo-Inositol, Naproxen TMS derivative, Uric acid, L-Kynurenine, L-Tryptophan 3TMS derivative, Stearic acid TMS derivative, Pseudo uridine penta-tms* |
| **Lipids Significant for COVID-19 Status** | *LPG17:2, SPBP18:1;O2, LPC4:0, ST24:4;O6, LPC3:1, PA26:4;O2, ST32:5;O10, PC(P-16:0/16:0), TG55:8, CE 18:1* |
| **Lipids Significant for Disease Severity (All Patients)** | *SPBP18:1;O2, PC6:0, CAR6:2;O2, LPI16:0, FA15:1;O, CAR18:2;O2, ST21:3;O3;GlcA, LPS19:1, PAO-29:0, PEO-38:7, PA36:3, PS42:2, Cer39:1;O4, CerP40:1;O2, PA36:2, Cer45:1;O4, HexCer36:2;O2, HexCer40:2;O2, TG55:4, PGO-40:3, PC43:0, Cer42:0;O, TG56:12, HexCer44:0;O2, PC.42.6_RT_23.452, PC.35.3_RT_23.583, Plasmenyl.PE.P.18.0_22.6_RT_23.611, TG53:2*  *Plasmenyl.PE.P.18.0_18.2_RT_23.828, TG54:8, Cer41:1;O4, ACer62:2;O3, TG58:2, ACer65:2;O4, Alkanyl.TG.O.16.0_16.0_16.0_RT_33.637, CE.18.0_RT_33.917* |
| **Lipids Significant for Disease Severity (Patients with COVID-19 only)** | *PC6:0, CAR6:2;O2, ST18:3;O2;GlcA, LPCO-17:0, LPS19:1, PAO-29:0, PS39:6, PA36:3, Cer34:2;O2, PA36:2, PA32:1, PA34:2, Cer45:1;O4, SM42:2;O2, HexCer40:2;O2, FA21:1;O, PI.38.6_RT_20.119, TG55:4, PE.16.0_18.3_RT_21.276, PC43:0, HexCer40:2;O2, Cer42:0;O, PC.35.3_RT_23.583, Plasmenyl.PE.P.18.0_18.2_RT_23.828, TG60:8, TG58:2, ACer65:2;O4, Alkanyl.TG.O.16.0_16.0_16.0_RT_33.637, CE.18.0_RT_33.917* |

**S3 Table**

**Molecules Selected from smCCA**

Lists of the molecules selected for smCCA for components 1 and 2.

| **Dataset** | **List of Molecules** |
| --- | --- |
| **Genes (Component 1)** | *ADHFE1, AGFG2, APOBEC3D, ARL4C, ATP10A, ATP1A3, BCL11B, BICDL1, C3orf18, CARNS1, CCDC65, CCL5, CD160, CD2, CD247, CD3D, CD3E, CD3G, CD6, CD8A, CDC25B, CLSTN1, CLUAP1, COQ10A, CRTAM, DCAF4, DLG3, DTX3, EDARADD, EID3, EOMES, EVL, FYN, GALNT6, GATA3, GGT7, GOLGA7B, GPR174, GPR68, GZMA, GZMK, IFFO2, IKZF3, ITGB7, KLRG1, KLRK1, LBH, LCK, LY9, MAN1C1, MCOLN2, MYBL1, NCALD, NCR3, NFATC2, NMT2, P2RY10, PJA1, PLCG1, PPP1R13B, PRKCQ, PYHIN1, PYROXD2, RARG, RFTN1, S100A12, S1PR5, SAMD3, SARM1, SBK1, SEMA4F, SH2D1A, SKAP1, SOX13, ST3GAL5, TBX21, TEF, TESPA1, TGFBR3, THEM4, TIGIT, TRAF5, TSEN54, UBASH3A, ZFYVE28, ZNF683, ZNF783, ZNF792* |
| **Proteins (Component 1)** | *S100A8, TNC, DEFA1* |
| **Metabolites (Component 1)** | *Creatinine 3TMS Derivative* |
| **Lipids (Component 1)** | *ST19:3;O3;S, FA18:4;O4, LPS22:2, LPC8:0, FA6:2;O6, ST21:4;O6, PS16:0, LPI13:0, ST27:4;O5, NeuAcHexCer38:1;O2, TG54:10, DG41:5, Cer42:1;O4, PE40:, PA34:2* |
| **Genes (Component 2)** | *ABI2, ABLIM1, ADGRL1, AKAP5, ALS2CL, AMOT, ANO8, ATP8B2, AUTS2, AZIN2, BCL2, C11orf95, C1orf21, C2CD2, CA11, CACNA2D2, CAMK2N1, CAMK4, CCDC136, CCL4, CD1C, CD300LB, CD40LG, CDHR3, CDK18, CDK20, CEP70, CLEC2D, COL17A1, CPAMD8, CYYR1, DCAF4, DGKE, DUSP14, DYRK2, ENKUR, ENPP5, FBXL16, FBXO41, FCRL3, FEZ1, FRMPD3, GPR183, HHAT, HOOK1, IL2RB, IL7R, INPP4B, ITPR3, ITPRIPL1, JHY, KLF12, KLRC4-KLRK1, LCN2, MAGEH1, MDFIC, MLLT3, MPP2, MPRIP, NECTIN3, NLRC3, NPR2.00, ORM2, OXNAD1, PAIP2B, PCSK5, PHLDB2, PLEKHA1, PLXDC1, PLXNA3, PPFIBP2, PPP1R16B, PRKACB, PROCR, PRSS23, PRXL2A, PTPN4, RASGRF2, RETN, RFPL2, RRAS2, RTL6, SCARB1, SGK1, SGSM1, SIDT1, SLAMF1, SLC25A53, SLC4A10, SLC7A6, SPON1, ST8SIA1, TBC1D4, TLR3, TMEM263, TRAF5, TSPOAP1, TTC24, TULP4, VSIG1, XCL2, ZC3H12B, ZC3H12D, ZNF202, ZNF239, ZNF286A, ZNF30, ZNF354C, ZNF365, ZNF781, ZNF792, ZNF831* |
| **Proteins (Component 2)** | *CST3, IGFBP2, LCN2* |
| **Metabolites (Component 2)** | *Quinolinic acid 2TMS derivative* |
| **Lipids (Component 2)** | *FA14:3;O2, ST19:3, LPS12:0, ST21:4;O6, PS16:0, LPI13:0, NeuAcHexCer38:1;O2,TG54:10, DG41:5* |

**S1 Fig.**

**Filtering data**

A summary of the two layer filtering process applied to the molecules which passed initial quality controls employed by Overmeyer et al.,


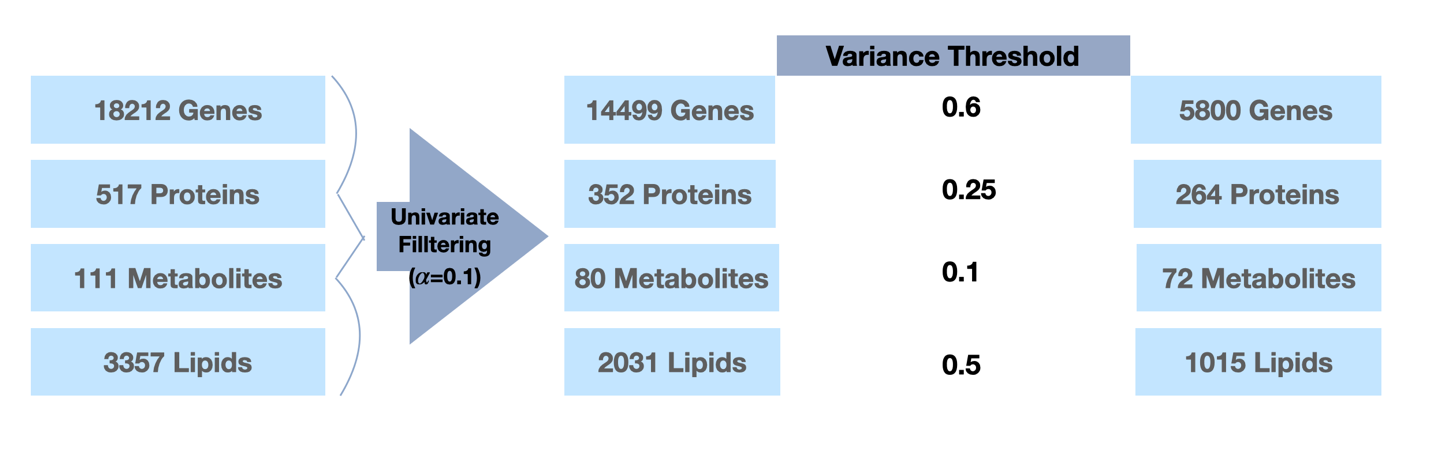


**S2 Fig.**

**Top 3 Genes for Covid-19 Status**

Violin Plots of the top 3 genes associated with COVID-19 status. These genes were selected via stability selection and univariate p-values were used to determine the 3 most significant.


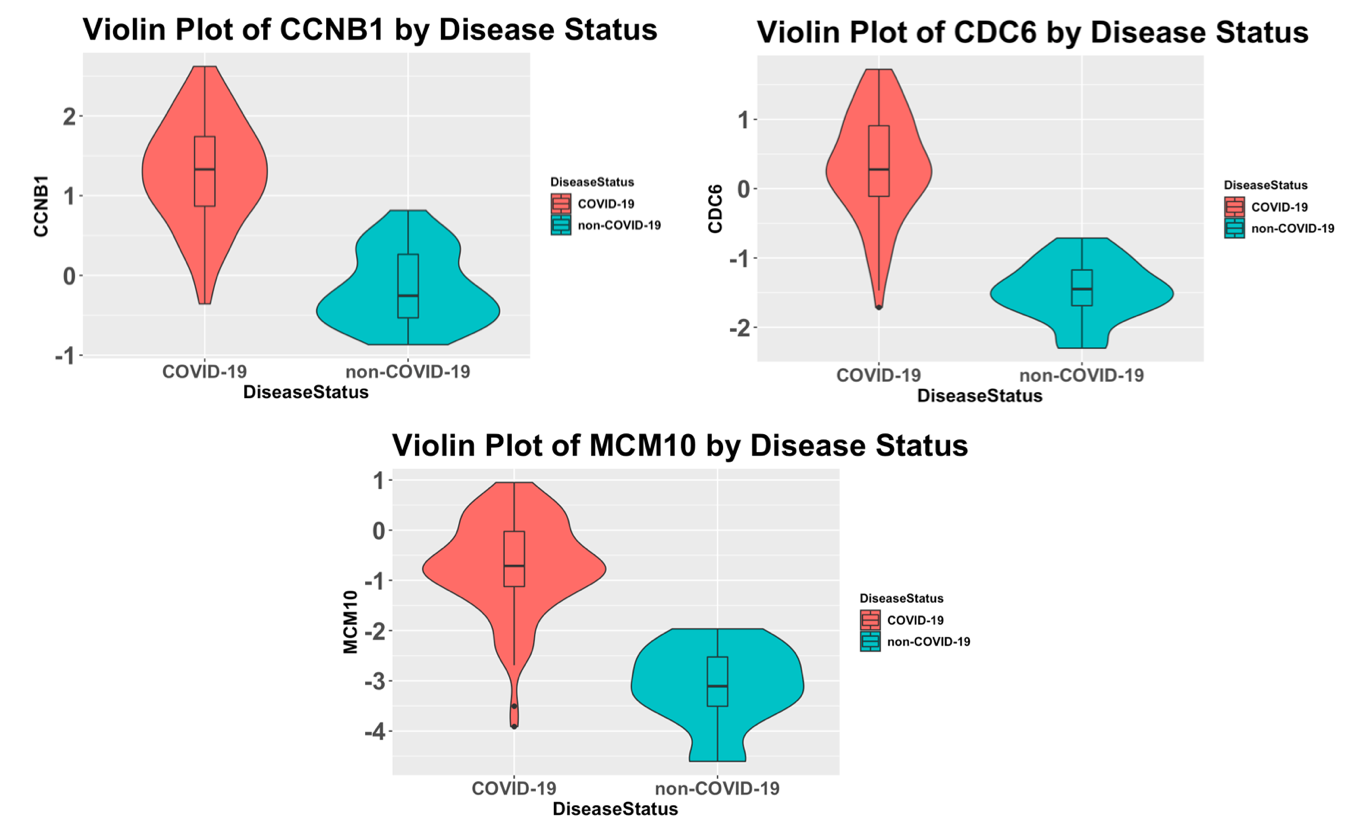


**S3 Fig.**

**Top 3 Proteins for Covid-19 Status**

Violin Plots of the top 3 proteins (by gene name) associated with COVID-19 status. These genes were selected via stability selection and univariate p-values were used to determine the 3 most significant.


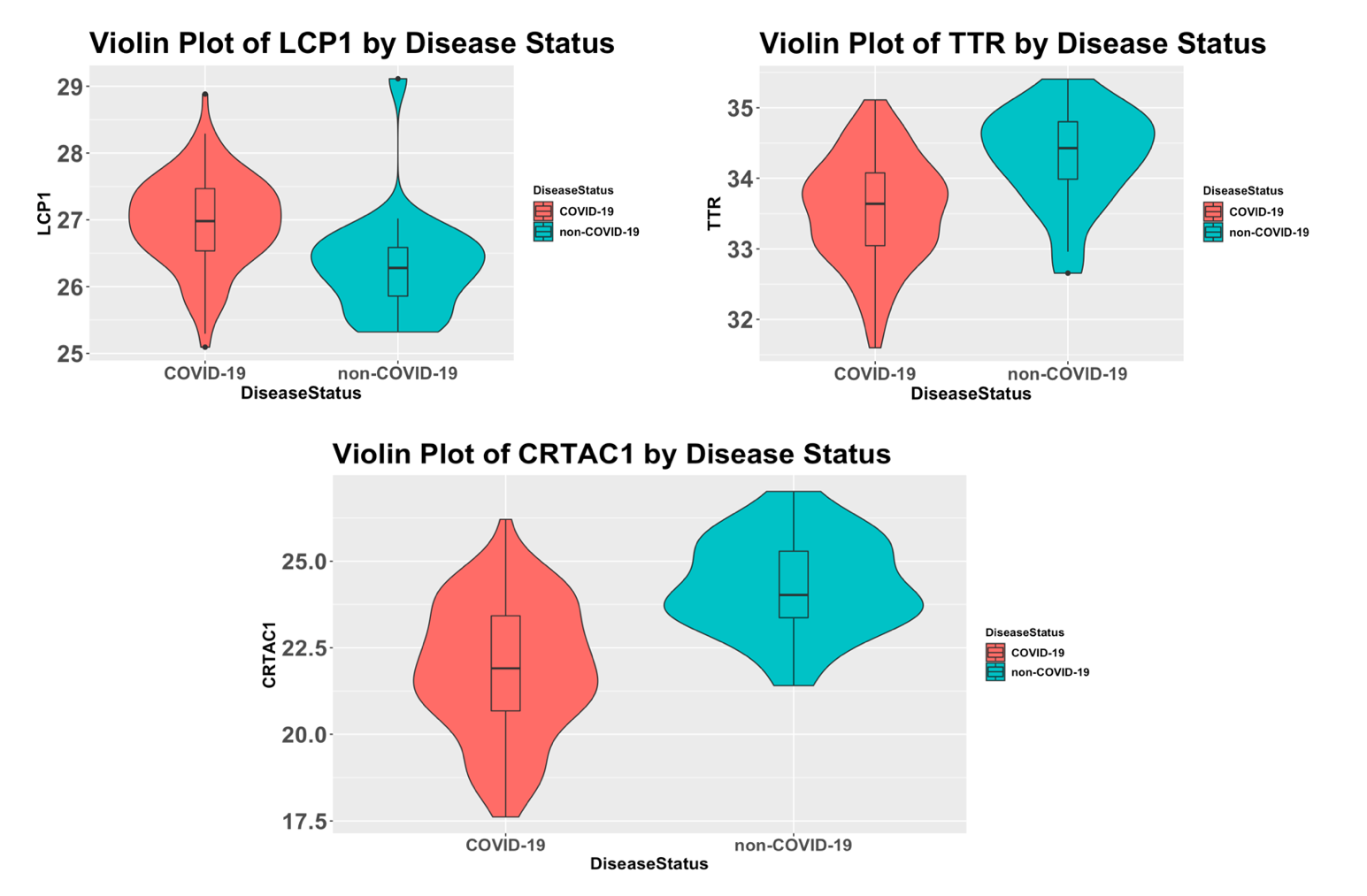


**S4 Fig.**

**PA34:2**

Violin Plots of PA34:2 by COVID-19 status

**
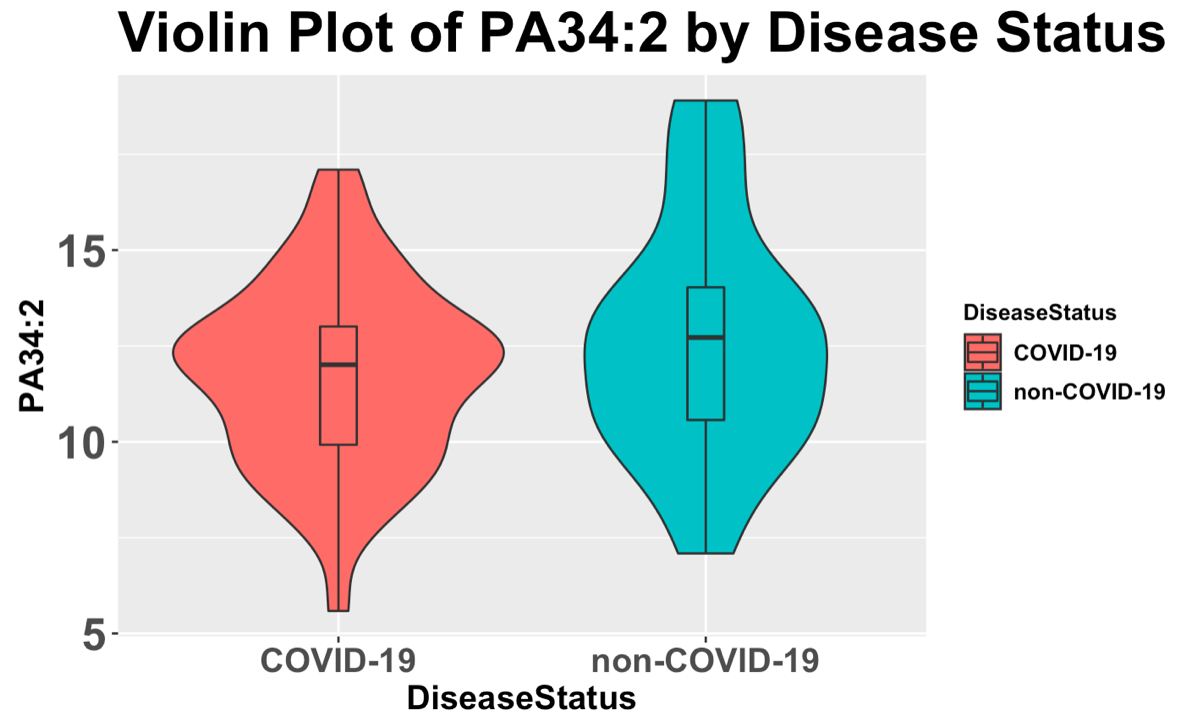
**

**S5 Fig.**

**Quinolinic acid 2TMS derivative**

Violin Plots of Quinolinic acid 2TMS derivative by COVID-19 status

**
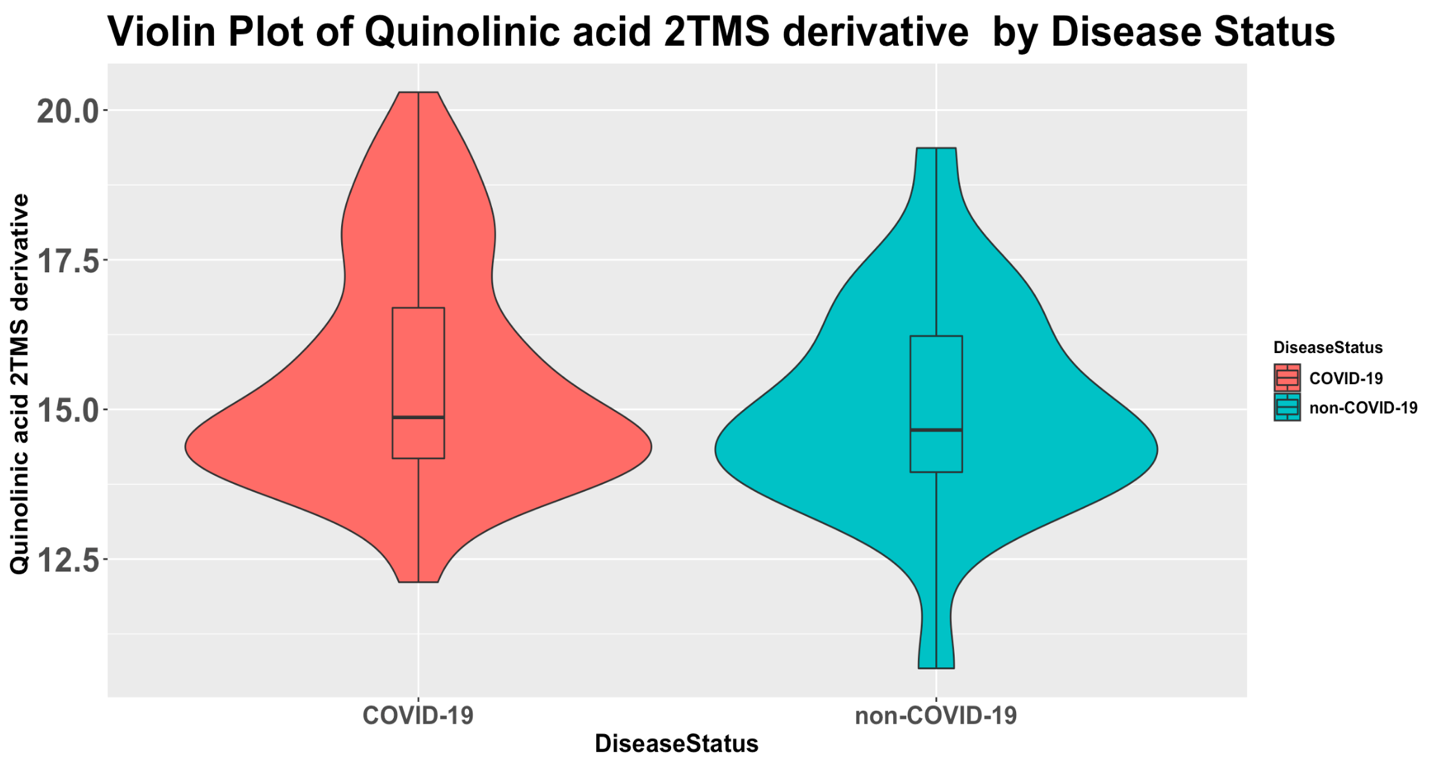
**

**S6 Fig.**

**LCN2 Protein**

Violin Plots of LCN2 Protein by COVID-19 status.

**
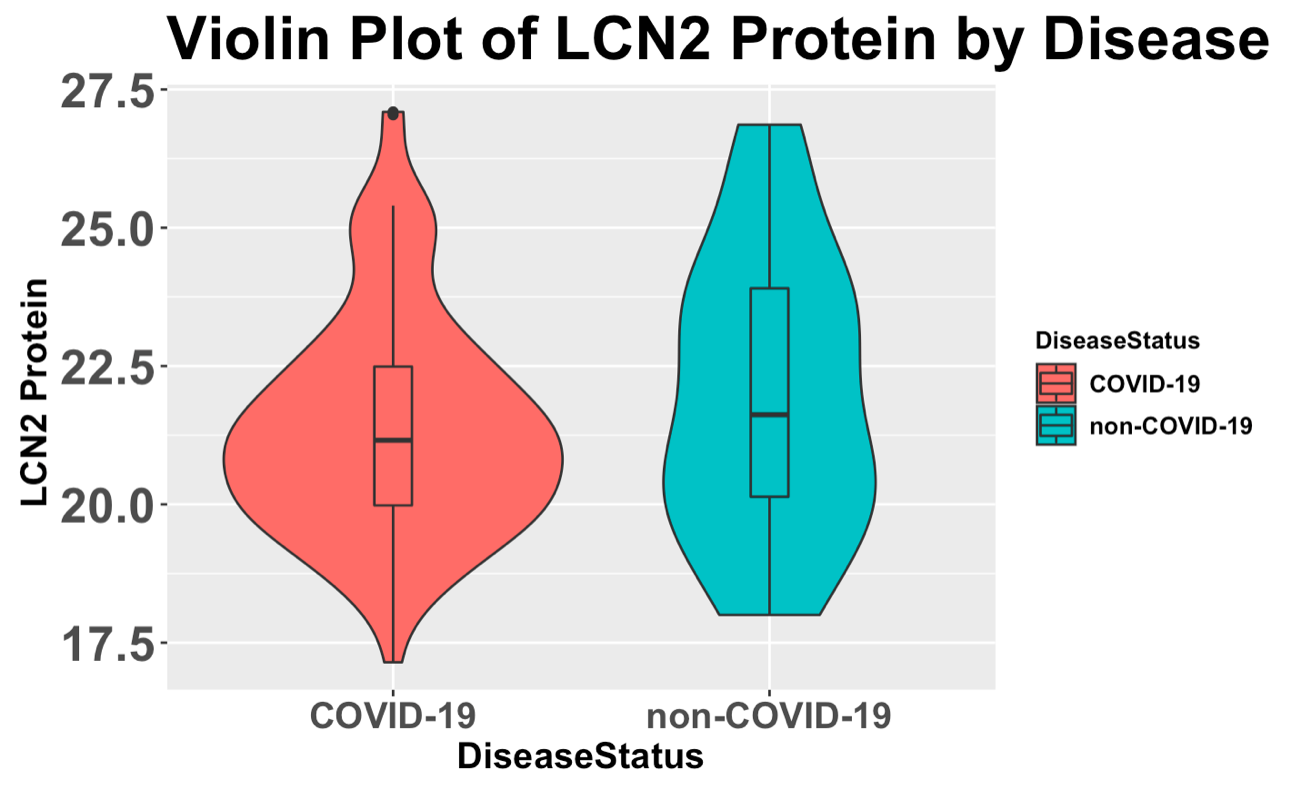
**

**S7 Fig.**

**Top 3 Genes smCCA Component 1**

Violin Plots of the top 3 genes (largest effect sizes) from component 1 of smCCA.

**
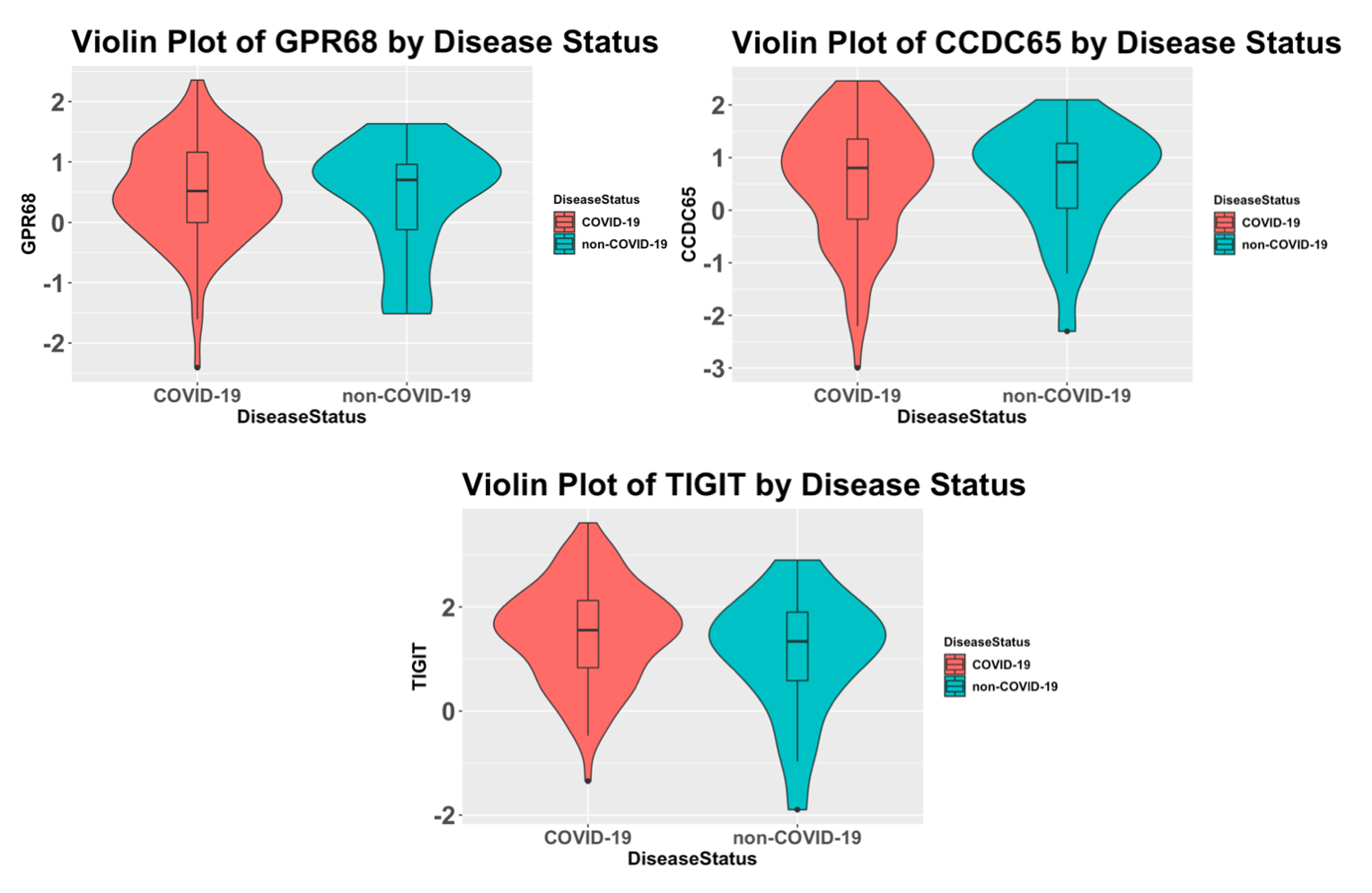
**

**S8 Fig.**

**Top 3 Genes smCCA Component 2**

Violin Plots of the top 3 genes (largest effect sizes) from component 2 of smCCA.

**
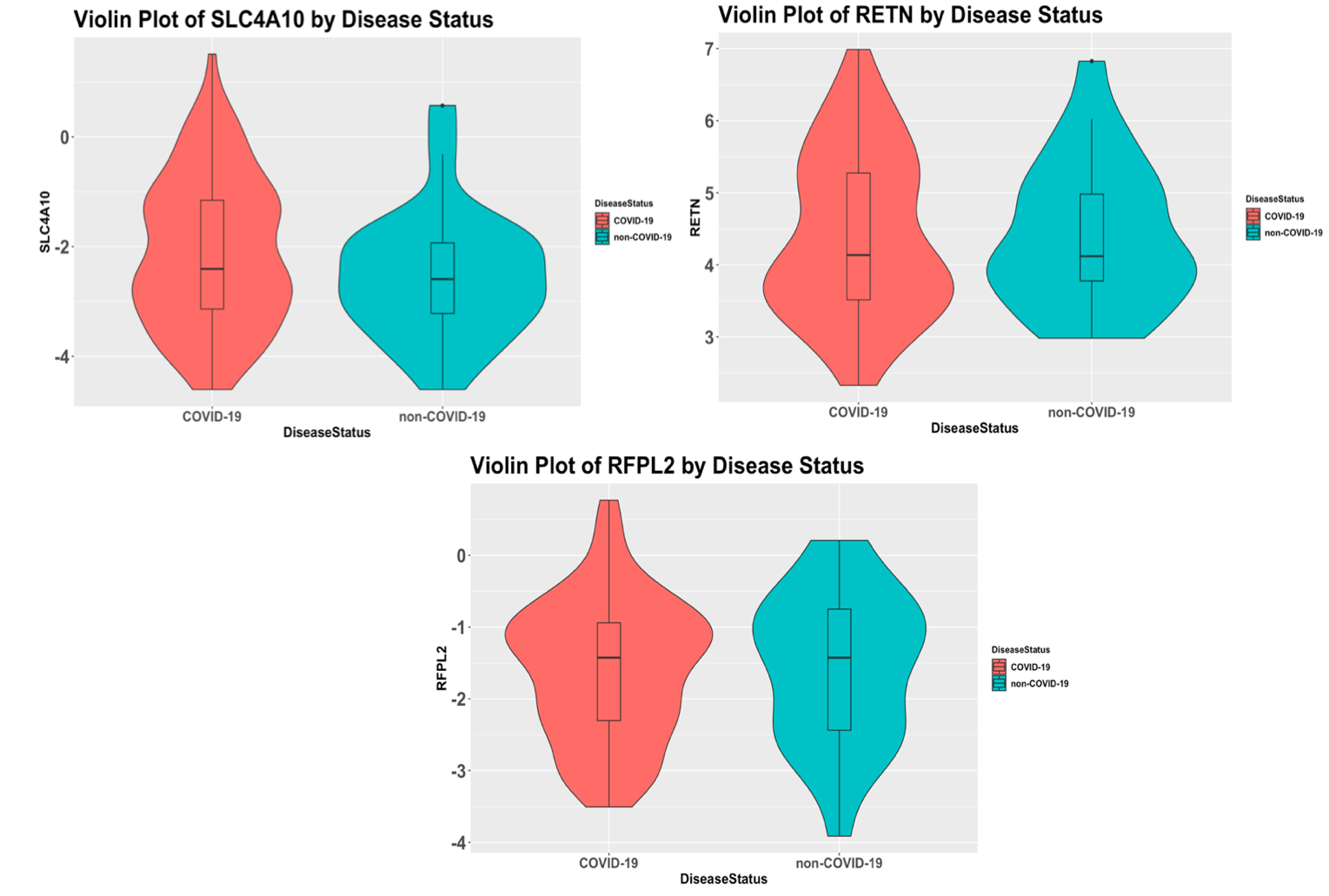
**
